# Probiotics for preventing neonatal sepsis in preterm neonates: a systematic review and meta-analysis for clinical practice

**DOI:** 10.4178/epih.e2025051

**Published:** 2025-09-03

**Authors:** Rizka Maulida, Radhian Amandito, Rinawati Rohsiswatmo, Amarila Malik

**Affiliations:** 1 Department of Epidemiology, Faculty of Public Health, Universitas Indonesia, Depok, Indonesia; 2 Pondok Indah General Hospital, Jakarta, Indonesia; 3 Division of Perinatology, Department of Pediatrics, Cipto Mangunkusumo Hospital/Faculty of Medicine, Universitas Indonesia, Jakarta, Indonesia; 4 Division of Pharmaceutical Microbiology and Biotechnology, Faculty of Pharmacy, Universitas Indonesia, Depok, Indonesia

**Keywords:** Late onset sepsis, Infant premature, Infection prevention, Microbiome therapy, Probiotics supplementation

## Abstract

Late-onset sepsis (LOS), occurring after 72 hours of birth, is a significant cause of morbidity and mortality especially in preterm neonates. Probiotics have been proposed as a preventive strategy to enhance gut health, modulate immune responses, and reduce the incidence of neonatal sepsis. We aimed to evaluate the effectiveness of probiotics in preventing neonatal sepsis in preterm neonates, with particular attention to the impact of different strains and dosage regimens. Eligible studies included preterm neonates (≤36 weeks gestational age) with culture-proven LOS and focused on probiotic supplementation. Comprehensive searches were conducted in MEDLINE via PubMed, Cochrane CENTRAL, Scopus, and ProQuest up to July 28, 2024. The Revised Cochrane Risk of Bias Tool (RoB 2.0) was applied to assess study quality, and a random-effects meta-analysis was performed using Review Manager version 5.4. Additionally, the certainty of the body of evidence was evaluated using the GRADE (Grading of Recommendations Assessment, Development, and Evaluation) approach. Thirty-one studies including 8,040 preterm neonates were reviewed. Meta-analysis demonstrated that probiotics significantly reduced the incidence of LOS (pooled risk ratio [RR], 0.83; 95% confidence interval [CI], 0.72 to 0.95). Greater efficacy was observed with multistrain formulations (RR, 0.76; 95% CI, 0.72 to 0.95) and low-dose regimens (RR, 0.72; 95% CI, 0.56 to 0.91). Probiotic supplementation was also associated with shorter hospital stays and a trend toward lower mortality, although the latter did not reach statistical significance. To effectively reduce LOS in preterm neonates, specific combinations of multistrain probiotics and optimized dosing strategies may provide the most benefit.

## GRAPHICAL ABSTRACT

[Fig f11-epih-47-e2025051]

## Key Message

• Late-onset sepsis (LOS) is a major cause of morbidity and mortality in preterm neonates, and the optimal approach for using probiotics to prevent LOS remains uncertain.

• Probiotics significantly reduce LOS risk (RR, 0.83; 95% CI, 0.72 to 0.95), with multistrain (RR, 0.76; 95% CI, 0.72 to 0.95) and low-dose formulations (RR, 0.72; 95% CI, 0.56 to 0.91) showing greater efficacy. Supplementation shortens hospital stays.

• These findings support the use in probiotics in neonatal care, as they enhance immune defense and reduce infection burden.

• This study informs clinical guidelines and highlights probiotics’ potential to improve neonatal outcomes and reduce healthcare costs.

## INTRODUCTION

Neonatal sepsis is a critical condition defined as bloodstream infection in infants younger than 28 days and remains a major global health challenge, particularly in resource-limited settings [1,2]. It is a leading contributor to neonatal morbidity and mortality. Late-onset sepsis (LOS), which occurs more than 72 hours after birth, is often caused by environmental pathogens introduced during or after delivery, including transmission via healthcare staff or medical equipment [3,4]. Invasive procedures and the frequent use of medical devices further heighten the risk of LOS, especially among preterm neonates. These infants are particularly susceptible due to underdeveloped immune defenses, immaturity of epithelial barriers, and frequent reliance on medical interventions such as catheters and feeding tubes [4]. The prevalence of LOS among preterm infants is high, with approximately 8.9% affected, corresponding to an incidence of 88.5 per 1,000 infants [5].

Comprehensive preventive strategies are essential to mitigate the burden of neonatal sepsis in preterm populations. Rigorous hand hygiene, including regular washing and use of alcohol-based sanitizers, plays a central role in preventing pathogen transmission. Early initiation of trophic enteral feeding supports gastrointestinal development and reduces the risk of bacterial translocation. Breastfeeding is strongly recommended, as it provides critical components such as immunoglobulin A, fatty acids, and amino acids that enhance immune defense. Despite these strategies, effective interventions with robust evidence remain urgently needed [6].

Probiotics have shown promise in fortifying the gut barrier, modulating immune responses through pathways involving TLR4 receptors and inflammatory cytokines, and preventing pathogenic colonization, thereby offering potential protection against neonatal sepsis [7]. Research indicates that preterm infants with LOS often display reduced microbial diversity and higher levels of pathogenic organisms, including Staphylococcus, prior to sepsis onset. Dysbiosis, defined by decreased microbial diversity, is particularly common in infants born before 32 weeks who later develop LOS. Thus, targeted modulation of the gut microbiota with probiotics may prevent dysbiosis and reduce the risk of sepsis, while simultaneously supporting healthy microbiota development during early life [8].

Recent evidence has confirmed the efficacy of probiotics in reducing the risk of necrotizing enterocolitis in preterm neonates. However, outcomes vary depending on probiotic strain, and no universally accepted protocols currently exist. This underscores the need for updated systematic reviews and meta-analyses to clarify their role in preventing LOS, especially with regard to optimal strains and dosages. Findings from such research could substantially influence clinical practice guidelines for managing neonatal sepsis and highlight the need for broader policies addressing structural and social inequities affecting neonatal health outcomes.

## MATERIALS AND METHODS

### Inclusion and exclusion criteria

We included studies involving preterm neonates (≤36 weeks gestational age) with culture-proven LOS that evaluated probiotic supplementation [8]. Studies were excluded if they involved term neonates, did not address sepsis, or used prebiotic or synbiotic interventions. The primary outcomes were neonatal sepsis and mortality, while the secondary outcome was the length of hospital stay or neonatal intensive care unit (NICU) stay.

### Search strategy

This review was prospectively registered in PROSPERO (ID: CRD42024581890). Comprehensive searches were conducted in major databases, including MEDLINE via PubMed, Cochrane CENTRAL, Scopus, and ProQuest. A structured search strategy was applied to identify studies published between January 2010 and July 28, 2024, limited to the English language. The timeframe (2010-2024) was chosen because 2010 marked the onset of more rigorous, large-scale probiotic trials for neonatal sepsis, notably the Probiotics in Preterm infantS (PiPS) trial [9]. Research from this period reflects contemporary clinical practices, improved trial methodologies, and enhanced safety assessments. Earlier studies were excluded due to methodological inconsistencies and limited relevance to current standards. Search terms were adapted for each database, focusing on combinations of keywords such as “preterm,” “probiotic,” “sepsis,” and “neonate.” Filters were applied to include only clinical trials and randomized controlled trials to ensure the selection of high-quality evidence. Details of the search process and the complete list of search terms are provided in Supplementary Material 1.

### Selection and data collection process

Two independent reviewers (RA, RM) performed the selection process. Titles and abstracts were first screened to identify potentially relevant studies, followed by manual removal of duplicates. Any disagreements were resolved through discussion or, when necessary, by consultation with a third reviewer (AM). Full texts of potentially eligible studies were retrieved and assessed against the inclusion criteria. Data extraction was independently performed by the same reviewers (RA, RM), focusing on study characteristics, participant demographics, intervention details, and outcome measures.

Studies were further categorized into subgroups based on probiotic strain and dosage. For dose-related subgrouping, interventions were classified as high dose (>10^9^ CFU/day) or low dose (≤10^9^ CFU/day). The cutoff was based on a previous randomized controlled trial comparing stool colonization in preterm infants given 3 different probiotic regimens [10]. In that trial, the low dose reflected the conventional dosage commonly used in NICUs, while the high dose represented an emerging practice increasingly adopted in recent studies due to evidence suggesting improved colonization and potential clinical benefits.

### Risk of bias assessment and synthesis methods

The Revised Cochrane Risk of Bias Tool (RoB 2.0) was used to assess the risk of bias in each included study. The tool evaluates 5 domains: bias in the randomization process, bias due to deviations from intended interventions, bias due to missing outcome data, bias in outcome measurement, and bias in selection of reported results. Risk was categorized as low, some concerns, or high. For dichotomous outcomes, risk ratios (RRs) with 95% confidence intervals (CIs) were calculated. For continuous outcomes, mean differences (MD) with 95% CIs were used. A random-effects meta-analysis was performed using Review Manager version 5.4 (Cochrane Collaboration, London, UK) to account for anticipated clinical heterogeneity across studies. When heterogeneity was minimal, a fixed-effects model was applied. Heterogeneity was assessed with the *I*^2^ statistic, with thresholds of 25% for low, 25-50% for moderate, and >50% for high heterogeneity. Statistical significance was set at a p-value <0.05. For studies not reporting data as mean±standard deviation (SD), conversion methods from Luo et al. [11] and Wan et al. [12] were applied.

Forest plots were generated to visually summarize meta-analytic findings, and funnel plots were used to assess publication bias. Subgroup and sensitivity analyses were conducted to explore sources of heterogeneity and test the robustness of results. Certainty of evidence was evaluated using the Grading of Recommendations Assessment, Development, and Evaluation (GRADE) approach, with GRADEpro software employed to generate summary-of-findings tables and facilitate quality grading. A random-effects model was applied for outcomes with significant clinical and methodological heterogeneity—particularly sepsis (including culture-proven, clinical, and composite forms) and LOS—due to variations in definitions and populations across studies. A fixed-effects model was used for outcomes reported more consistently, such as mortality. Secondary outcomes, such as hospital stay duration, were analyzed descriptively because of variability in reporting formats (mean±SD, median with interquartile range, or range), and the lack of standardized statistical comparisons. Reporting adhered to Preferred Reporting Items for Systematic Reviews and Meta-Analyses (PRISMA) 2020 guidelines.

### Ethics statement

Ethical approval from the institutional research ethics committee was not required as this systematic review gathers data from previously published data and did not involve human subjects directly.

## RESULTS

A total of 31 studies out of 177 met the inclusion criteria and were included in this review [9,10,13-41] (Supplementary Material 1). These comprised randomized controlled trials (RCTs) and non-randomized controlled trials (NRCTs) that investigated the use of probiotics in preterm neonates with sepsis. Supplementary Material 2 provides comprehensive details on study design, participant demographics, probiotic strains used, dosage and duration of interventions, and reported primary and secondary outcomes.

Risk of bias was assessed for each study using the Revised Cochrane Risk of Bias Tool (RoB 2.0), with results summarized in Supplementary Material 3. Overall, the risk of bias varied: some studies demonstrated low risk, while others showed concerns in 1 or more domains, most notably randomization and blinding. Supplementary Material 4 presents a summary of findings for probiotics compared with control interventions, evaluated using the GRADE approach. Certainty of evidence ranged from moderate to very low depending on outcome and formulation. For LOS, certainty was moderate, with probiotics significantly reducing risk (RR, 0.83; 95% CI, 0.72 to 0.95), equivalent to 141 fewer cases per 1,000 neonates relative to controls. Subgroup analyses for specific strains, including *Lactobacillus, Bifidobacterium*, and *Saccharomyces*, also demonstrated moderate certainty but varied in effectiveness. Downgrading occurred primarily due to inconsistency and imprecision. For mortality, the overall certainty was moderate but was downgraded for certain strains and doses. For length of hospital stay, the quality of evidence was low to very low, reflecting substantial heterogeneity and imprecision.

### Late-onset sepsis

**Figure 1.**
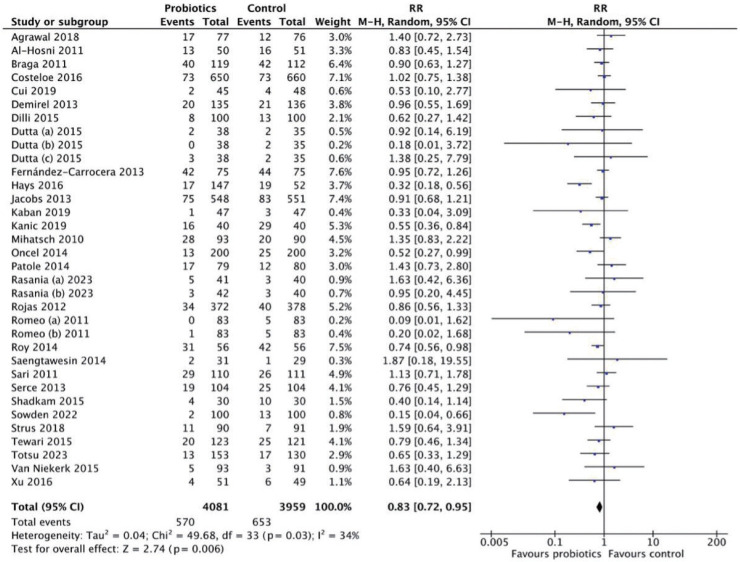
Forest plot analysis for late-onset sepsis incidence. RR, risk ratio; M-H, Mantel-Haenszel; CI, confidence interval; df, degrees of freedom.

The meta-analysis included 8,040 preterm neonates (4,081 in the probiotics group and 3,959 in the control group) (Figure 1). The pooled RR was 0.83 (95% CI, 0.72 to 0.95), indicating a significant reduction in LOS incidence among neonates receiving probiotics compared with controls. Most studies reported lower sepsis rates in probiotic groups, with CIs generally not crossing the line of no effect, supporting consistency in probiotic benefit. Heterogeneity was moderate (*I*^2^=34%).

**Figure 2.**
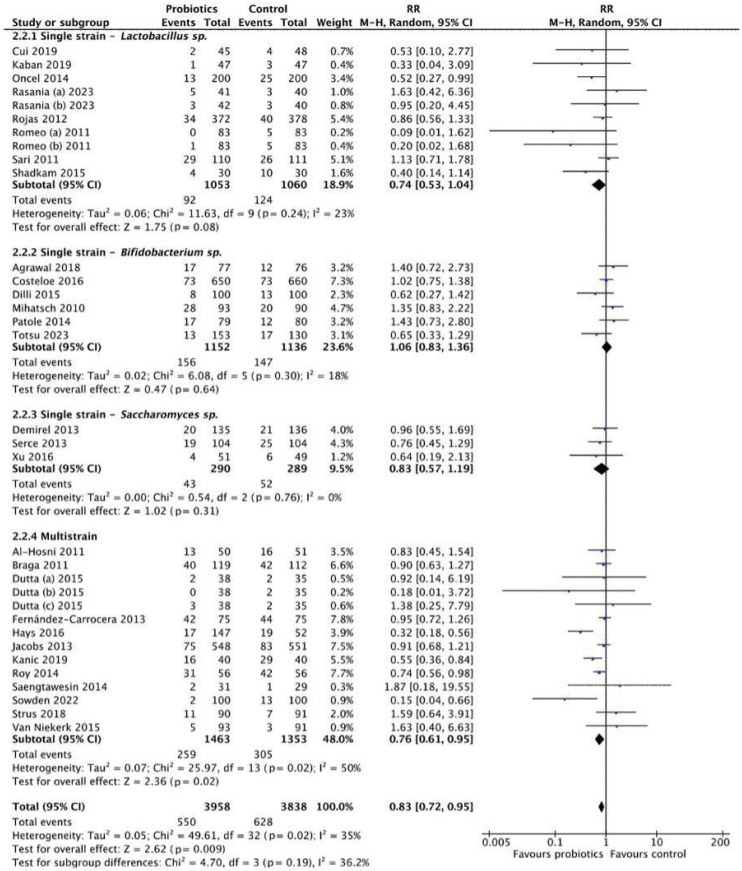
Subgroup analysis for late-onset sepsis incidence by strain. RR, risk ratio; M-H, Mantel-Haenszel; CI, confidence interval; df, degrees of freedom.

Effectiveness was further assessed by probiotic strain (Figure 2). Multistrain formulations demonstrated the strongest benefit (RR, 0.76; 95% CI, 0.72 to 0.95; *I*^2^=50%; p=0.02).

**Figure 3.**
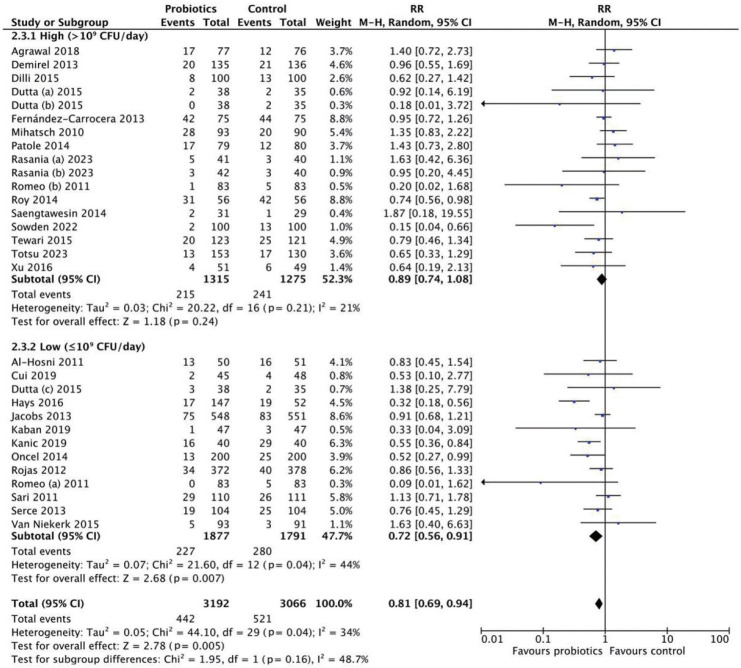
Subgroup analysis for late-onset sepsis incidence by dosage. RR, risk ratio; M-H, Mantel-Haenszel; CI, confidence interval; df, degrees of freedom.

Subgroup analysis by dosage classified interventions as high dose (>10^9^ CFU/day) or low dose (≤10^9^ CFU/day) (Figure 3). The high-dose subgroup showed no significant reduction (RR, 0.89; 95% CI, 0.74 to 1.08; p=0.24), with low heterogeneity (*I*^2^=21%). Conversely, the low-dose subgroup demonstrated a significant reduction in LOS (RR, 0.72; 95% CI, 0.56 to 0.91; p=0.007), with moderate heterogeneity (*I*^2^=44%). These findings suggest that probiotics, particularly at lower doses, are associated with a reduced risk of LOS.

**Figure 4.**
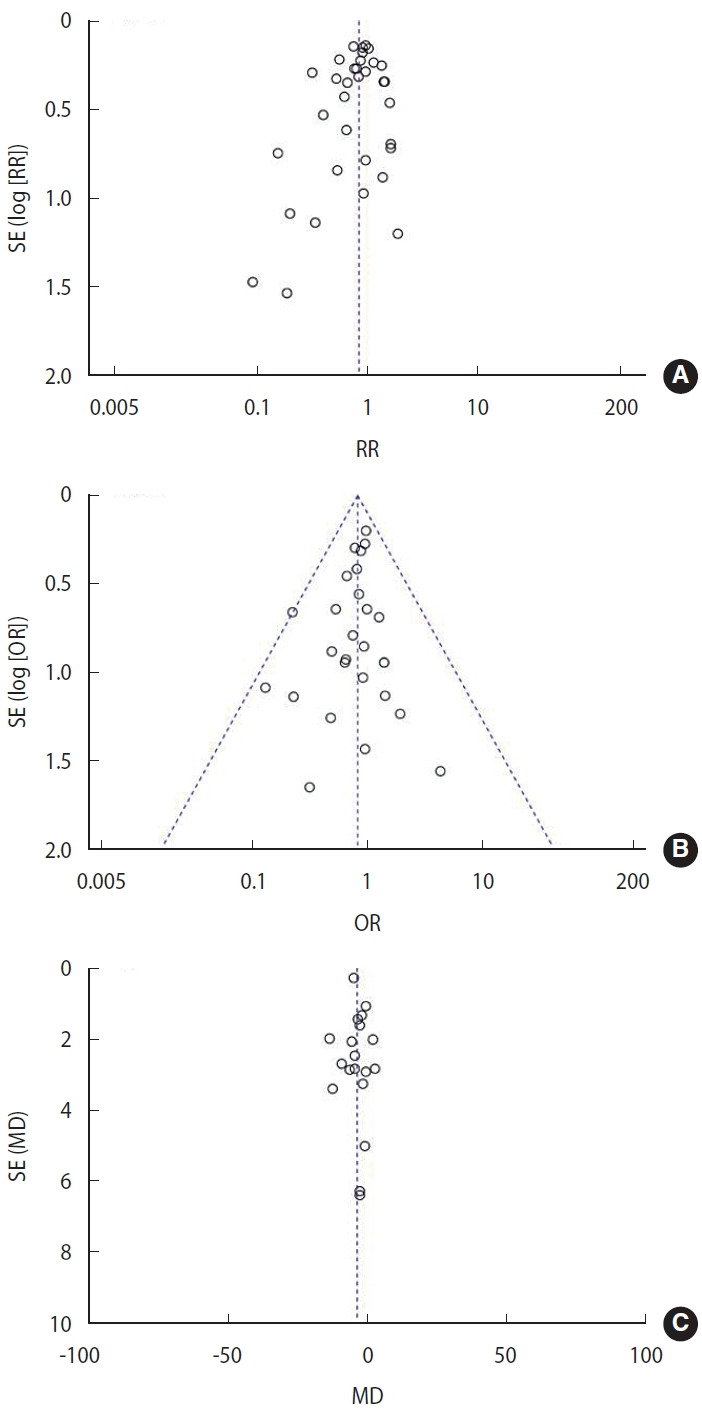
Funnel plot analysis. (A) For late-onset sepsis incidence, (B) for mortality, and (C) for length of stay. SE, standard error; RR, risk ratio; OR, odds ratio; MD, mean difference.

The funnel plot showed a relatively symmetrical distribution of effect sizes around the pooled estimate, with most studies clustered toward the top, indicating low standard errors (Figure 4A).

### Mortality

**Figure 5.**
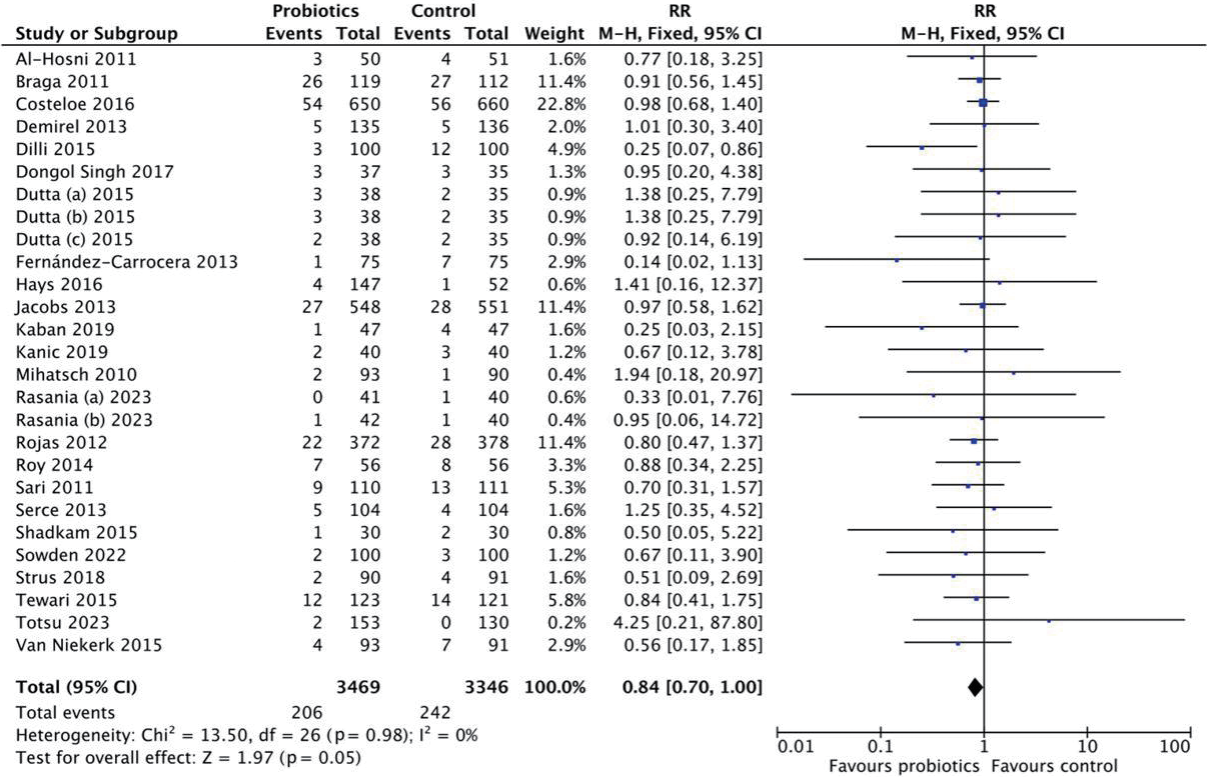
Forest plot analysis for mortality. RR, risk ratio; M-H, Mantel-Haenszel; CI, confidence interval; df, degrees of freedom.

**Figure 6.**
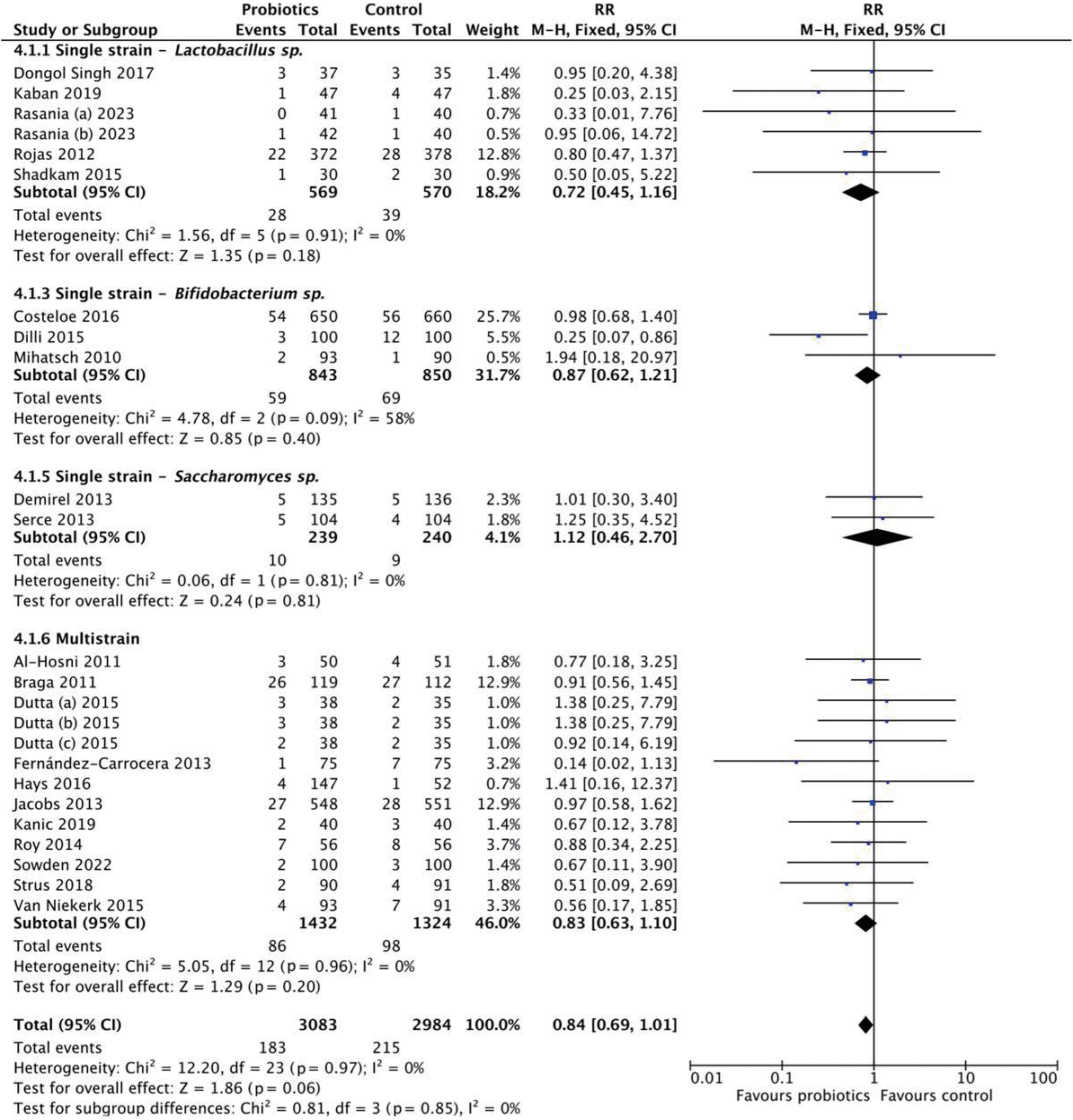
Subgroup analysis for mortality by strain. RR, risk ratio; M-H, Mantel-Haenszel; CI, confidence interval; df, degrees of freedom.

**Figure 7.**
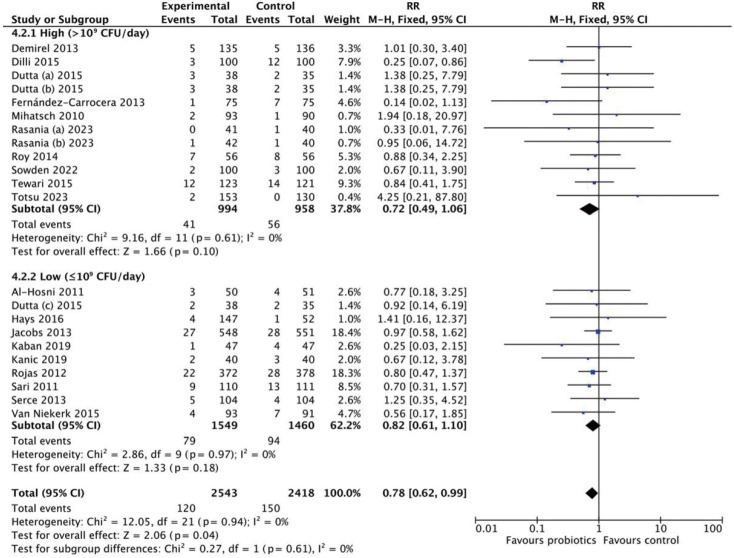
Subgroup analysis for mortality by dosage. RR, risk ratio; M-H, Mantel-Haenszel; CI, confidence interval; df, degrees of freedom.

This analysis included 6,815 neonates (3,469 in the probiotics group and 3,346 in the control group) (Figure 5). The pooled RR was 0.84 (95% CI, 0.70 to 1.00), with no heterogeneity (*I*^2^=0%). These findings suggest no statistically significant reduction in mortality (p=0.05). Subgroup analyses by strain and dosage were also conducted (Figure 6). Neither *Lactobacillus* (RR, 0.72; 95% CI, 0.45 to 1.16) nor *Bifidobacterium* (RR, 0.87; 95% CI, 0.62 to 1.21) demonstrated significant effects. Multistrain probiotics produced a pooled RR of 0.83 (95% CI, 0.63 to 1.10), also non-significant. For dosage, high-dose probiotics (>10^9^ CFU/day) yielded an RR of 0.72 (95% CI, 0.49 to 1.06), while low-dose probiotics (≤10^9^ CFU/day) had an RR of 0.82 (95% CI, 0.61 to 1.10) (Figure 7).

The funnel plot (Figure 4B) showed approximate symmetry around the pooled effect, with clustering at the top reflecting low standard errors and precise estimates. Some asymmetry at the bottom, where smaller studies displayed greater variability, suggests potential publication bias. However, overall symmetry indicates that publication bias is unlikely to significantly influence conclusions.

### Length of stay

**Figure 8.**
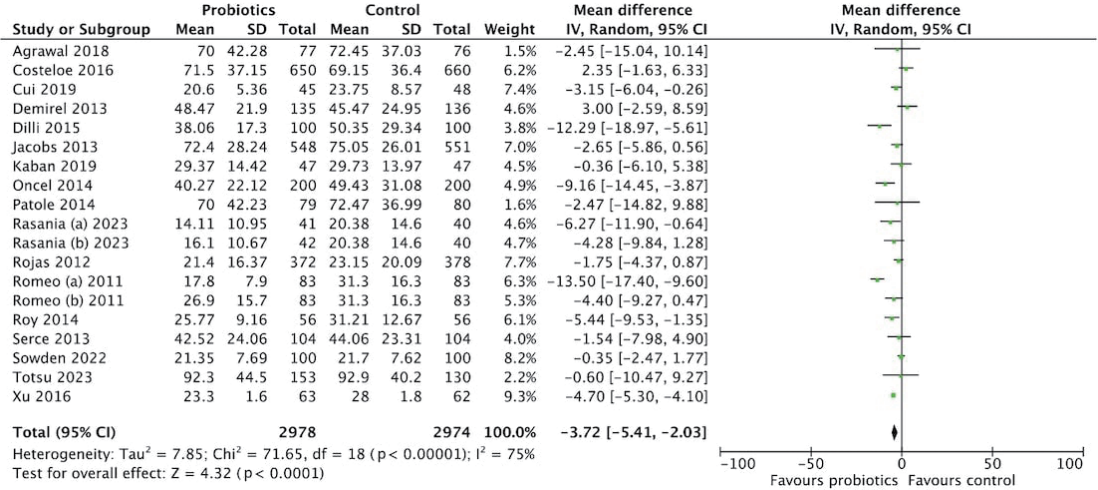
Forest plot analysis for length of stay. SD, standard deviation; CI, confidence interval; df, degrees of freedom.

**Figure 9.**
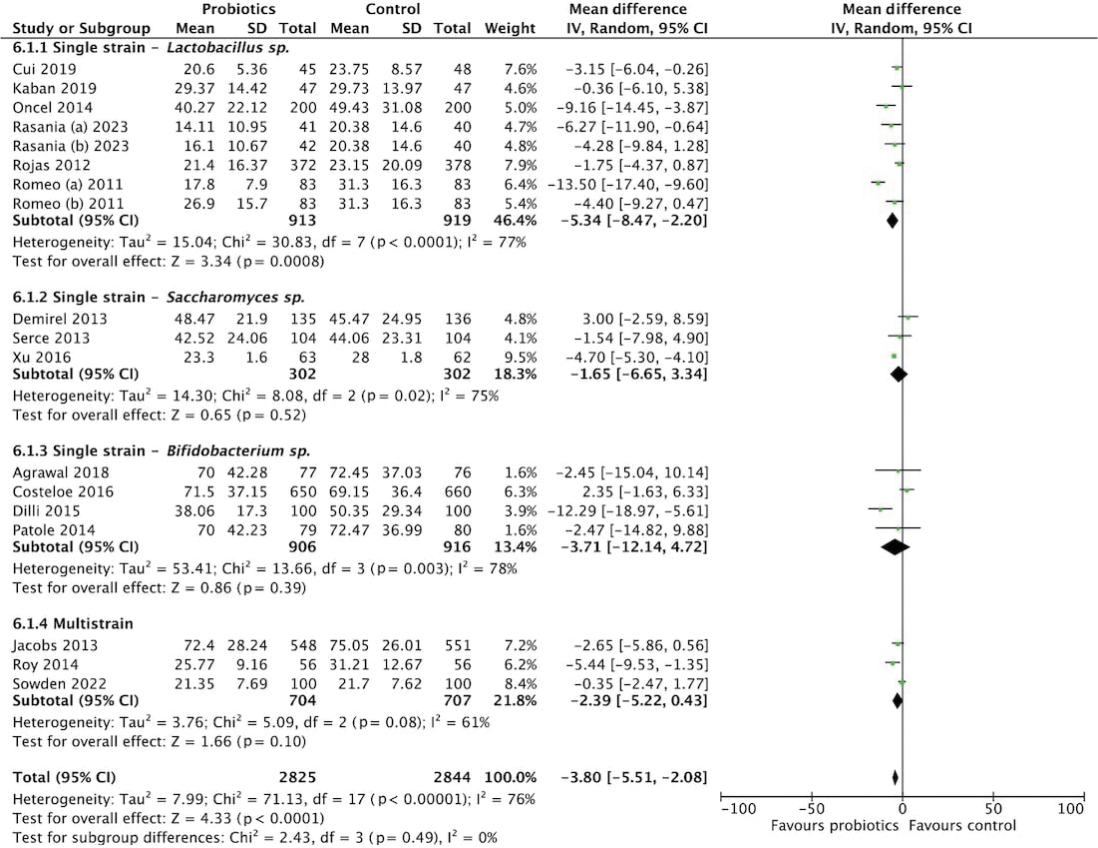
Subgroup analysis for length of stay by strain. SD, standard deviation; CI, confidence interval; df, degrees of freedom.

**Figure 10.**
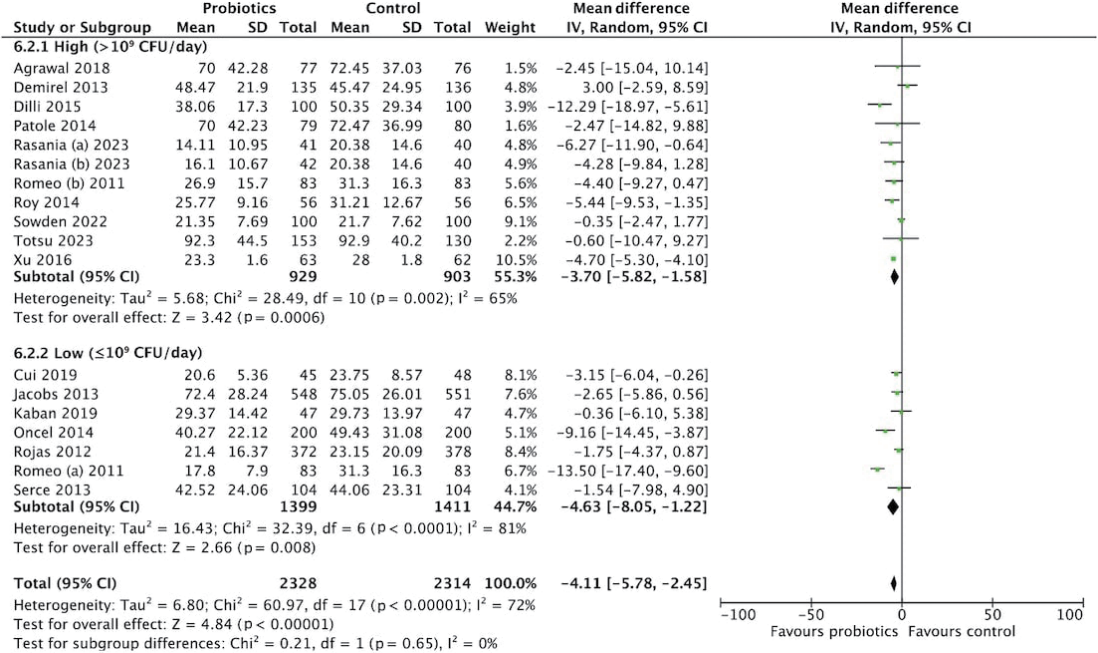
Subgroup analysis for length of stay by dosage. SD, standard deviation; CI, confidence interval; df, degrees of freedom.

The meta-analysis of 5,952 participants (2,978 in the probiotics group and 2,974 in the control group) assessed the effect of probiotics on hospital length of stay (Figure 8). The pooled MD was -3.72 days (95% CI, -5.41 to -2.03), favoring probiotics. Substantial heterogeneity was observed (*I*^2^=75%, p<0.001). Strain-specific subgroup analysis showed variable effects: *Lactobacillus* reduced length of stay by -5.34 days (95% CI, -8.47 to -2.20), *Bifidobacterium* by -3.71 days (95% CI, -12.14 to 4.72), and multistrain formulations by -2.39 days (95% CI, -5.22 to 0.43) (Figure 9). Dosage analysis revealed greater benefit with the low-dose group (≤10^9^ CFU/day), showing a MD of -4.63 days (95% CI, -8.05 to -1.22) compared with the high-dose group (>10^9^ CFU/day), which showed -3.70 days (95% CI, -5.82 to -1.58) (Figure 10). These findings suggest that probiotic supplementation, particularly at lower doses, may reduce hospital stay duration.

The funnel plot was generated to assess potential publication bias in the meta-analysis. The plot showed a generally symmetrical distribution of studies around the mean difference, with most studies clustering near the top, indicating lower standard errors and more precise estimates (Figure 4C).

## DISCUSSION

This analysis demonstrated that probiotics are effective in reducing the incidence of late-onset LOS in preterm neonates. The effect was most pronounced with multistrain probiotics and lower-dose formulations, suggesting that specific strain combinations and optimized dosing strategies may provide the greatest benefit. The superior performance of lower-dose regimens in reducing LOS and shortening hospital stay may be explained by the vulnerability of the immature gut environment. High doses, while seemingly more potent, may overwhelm neonatal gut microbiota, leading to less effective colonization or even disruption of microbial balance [42]. Subgroup analysis confirmed a significant reduction in sepsis with low-dose probiotics (RR, 0.72; 95% CI, 0.56 to 0.91), with moderate heterogeneity (*I*^2^=44%). This heterogeneity likely reflects differences in study populations, probiotic strains, timing and duration of administration, and baseline sepsis risk across settings. Although probiotics were associated with a trend toward lower mortality, this did not reach statistical significance. Therefore, caution remains warranted when considering higher doses.

Probiotic supplementation was also linked to a reduction in hospital length of stay, highlighting its potential to accelerate recovery in preterm neonates. The benefit was greater with lower doses, underscoring the importance of dose optimization for therapeutic success. Differences in effectiveness among probiotic strains further indicate that not all strains confer equal benefits. Some appear more effective than others in improving clinical outcomes, an observation with direct implications for neonatal care protocols.

Among tested strains, *Lactobacillus* species showed the strongest effects. These bacteria are among the earliest colonizers of the human gut and are well known for producing lactic acid, which lowers intestinal pH and inhibits pathogenic growth [43]. In addition, *Lactobacillus* enhances gut barrier integrity, reducing bacterial translocation into the bloodstream, a critical factor in preventing sepsis. Their immunomodulatory properties, including stimulation of anti-inflammatory cytokines, may also contribute to infection prevention in preterm infants. Collectively, these characteristics make *Lactobacillus* particularly valuable in neonatal care [44].

The results of our meta-analysis are consistent with findings from previous systematic reviews and clinical studies. A Cochrane review on probiotics for the prevention of necrotizing enterocolitis in preterm infants reported a significant reduction in severe necrotizing enterocolitis (stage II-III) as well as a substantial decrease in all-cause neonatal mortality with probiotic supplementation [45]. Similarly, Deshpande et al. [46] demonstrated that probiotics reduced the incidence of LOS with a number needed to treat (NNT) of 25 to prevent 1 case of sepsis, and also decreased the risk of death with an NNT of 50 to prevent 1 death. A 2016 meta-analysis further supported these findings, showing that enteral probiotic supplementation significantly reduced the risk of any sepsis (RR, 0.83; 95% CI, 0.73 to 0.94) with low heterogeneity (*I*^2^=26%) [46]. This review also highlighted reductions in bacterial sepsis (RR, 0.82; 95% CI, 0.71 to 0.95) and fungal sepsis (RR, 0.57; 95% CI, 0.41 to 0.78), both with no significant heterogeneity (*I*^2^=0%). Importantly, the benefits were evident even in very low birth weight infants, reinforcing the role of probiotics as a preventive strategy in this particularly vulnerable population [47].

The findings from this meta-analysis underscore the effectiveness of probiotics in reducing neonatal sepsis, particularly LOS, in preterm infants. Across studies, probiotics consistently lowered sepsis rates, a noteworthy outcome given the high infection risk in this group. However, the analysis also shows that probiotic efficacy is not uniform; outcomes appear to be highly strain-specific and dose-dependent. Multistrain formulations were particularly effective, suggesting potential synergistic effects that improve gut colonization, immune modulation, and competitive exclusion of pathogens. The evidence favoring multistrain or low-dose formulations has direct implications for neonatal care, where probiotics could be integrated into NICU protocols as a preventive intervention. Such integration may reduce sepsis incidence, improve survival, and support better overall health outcomes, aligning with global health priorities and sustainable development goals through the establishment of evidence-based clinical guidelines.

Despite these promising results, this meta-analysis has several limitations that must be acknowledged. One important limitation was the inability to stratify outcomes by intervention duration or by gestational age (very preterm vs. preterm), due to the limited number of studies available in these subgroups. Consequently, potential differences in probiotic effectiveness based on treatment duration or degree of prematurity remain uncertain. Language restrictions also excluded non-English publications, which may have limited the comprehensiveness of the evidence base. Additionally, the GRADE assessment indicated moderate certainty for most outcomes, such as the reduction in LOS, but downgrades were applied for inconsistency and imprecision in several subgroup analyses. For mortality and hospital length of stay, certainty was lower, ranging from moderate to very low, reflecting variability in study design, outcome reporting, and sample sizes. These findings highlight the need for future studies with broader inclusion criteria, more granular stratification by gestational age and intervention duration, and standardized probiotic formulations. Such research would strengthen the evidence base and provide clearer, more robust guidance for optimizing probiotic use in neonatal care.

The findings highlight the strain-specific and dose-dependent nature of probiotic benefits, with *Lactobacillus* strains showing particular promise in enhancing gut health and preventing infections. These results suggest that optimizing probiotic regimens could substantially improve neonatal outcomes, particularly within NICU settings. Nonetheless, important limitations remain, including the scarcity of data on very preterm infants and variability in the duration of probiotic administration. In addition, differences in NICU conditions across countries—such as sepsis prevention protocols, resuscitation practices, and nutritional support—may also influence study outcomes. Further research is needed to clarify whether previously identified effective strains were consistently included in trials and to determine which multistrain probiotic combinations provide the strongest evidence base. Despite these limitations, integrating probiotics into standard neonatal care protocols represents a promising strategy for improving both survival and overall health in preterm infants.

## CONCLUSION

Optimizing probiotic regimens holds significant promise for improving health outcomes in neonates, particularly those admitted to NICUs. Probiotic supplementation in these vulnerable infants has been associated with shorter hospital stays, suggesting faster recovery and fewer complications, as well as a trend toward reduced mortality. To maximize these therapeutic benefits, emphasis should be placed on the use of specific combinations of multistrain probiotics, alongside carefully optimized dosing strategies to achieve the best clinical results. Tailoring the selection of probiotic strains and dosing protocols to the unique needs of preterm neonates is likely to yield the greatest improvements in neonatal outcomes within NICU settings.

## Figures and Tables

**Figure f11-epih-47-e2025051:**
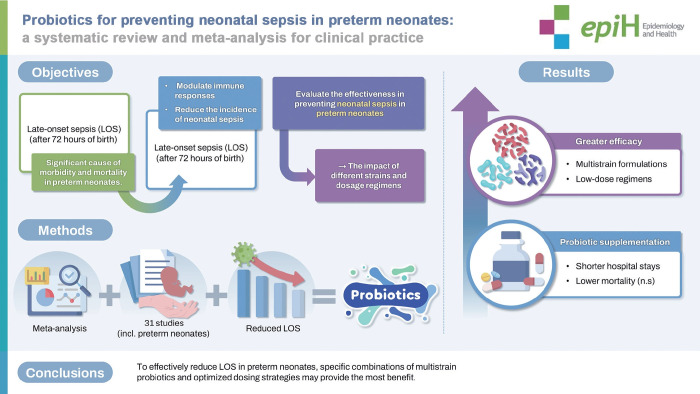


## Supplementary materials

Supplementary Material 1. Search strategy according to PRISMA Article Search 2020

Supplementary Material 2. Study characteristics and outcomes measured

Supplementary Material 3. The Revised Cochrane Risk of Bias Tool (RoB 2.0) assessment

Supplementary Material 4. Summary of findings and certainty assessment (GRADE)
