# Supplementary material for: Probiotics for preventing neonatal sepsis in preterm neonates: a systematic review and meta-analysis for clinical practice
Source: Epidemiol Health. 2025 Sep 3;47:e2025051. doi: 10.4178/epih.e2025051 (PMC12869123; doi:10.4178/epih.e2025051)
Supplement: Supplementary Material 1. — Search strategy according to PRISMA Article Search 2020 [file epih-47-e2025051-Supplementary-1.docx]

**Supplementary Material 1.** Search strategy according to PRISMA Article Search 2020

**Identification of studies via other methods**

**Identification of studies via databases and registers**

Records identified from:

Citation searching (n = 6)

Records removed *before screening*:

Duplicate records removed (n = 72)

Records marked as ineligible by automation tools (year ineligible) (n = 7)

Records identified from*:

MEDLINE via PubMed

(n = 47)

Cochrane CENTRAL

(n = 176)

Scopus (n = 3)

ProQuest (n = 30)

**Identification**

Records screened

(n = 177)

Records excluded**

(n = 135)

Reports not retrieved

(n = 0)

Reports sought for retrieval

(n = 6)

Reports sought for retrieval

(n = 42)

Reports not retrieved (full-text unavailable)

(n = 11)

**Screening**

Reports excluded (n = 0)

Reports assessed for eligibility

(n = 6)

Reports assessed for eligibility

(n = 31)

Reports excluded:

Non-english (n = 2)

No sepsis analysis (n = 1)

Retracted paper (n = 1)

Cohort (n = 1)

Intervention includes prebiotic (n = 1)

Studies included in review

(n = 31)

**Included**

**MEDLINE via PubMed (n = 47)**

**Filters applied: clinical trial, randomized controlled trial, year filter (2010 onwards)**

(Preterm[Title/Abstract] OR premature[Title/Abstract]) AND (Infection[Title/Abstract] OR sepsis[Title/Abstract] OR septicemia[Title/Abstract] OR septicaemia[Title/Abstract] OR bacteremia[Title/Abstract] OR bacteraemia[Title/Abstract] OR septic shock[Title/Abstract]) AND (Probiotic[Title/Abstract] OR Lactobacilli[Title/Abstract] OR Lactobacillus[Title/Abstract] OR Bifidobacterium[Title/Abstract])

**Cochrane CENTRAL (n = 176)**

**Filters applied: with Publication Year from 2010 to 2024, in Trials**

#1 preterm 18530

#2 premature 24131

#3 #1 OR #2 32263

#4 Infection 118146

#5 sepsis 15523

#6 septicemia 1260

#7 septicaemia 1260

#8 bacteremia 3191

#9 bacteraemia 3191

#10 septic shock 4404

#11 #4 OR #5 OR #6 OR #7 OR #8 OR #9 OR #10 130344

#12 Probiotic 7766

#13 Lactobacilli 1222

#14 Lactobacillus 6939

#15 Bifidobacterium 3993

#16 #12 OR #13 OR #14 OR #15 12285

#17 #3 AND #11 AND #16 176

**Scopus (n = 3)**

TITLE-ABS-KEY ( ( preterm OR premature ) AND ( infection OR sepsis OR septicemia OR bacteremia OR septicaemia OR bacteraemia OR septic AND shock ) AND ( probiotic OR lactobacilli OR lactobacillus OR bifidobacterium ) ) AND ( LIMIT-TO ( DOCTYPE , "ar" ) )

**ProQuest (n = 30)**

**Additional limits - Date: From 2010 to 2024, Document type: Article; Language: English;**

abstract((Preterm OR premature) AND (Infection OR sepsis OR septicemia OR bacteremia OR septicaemia OR bacteraemia OR septic shock) AND (Probiotic OR Lactobacilli OR Lactobacillus OR Bifidobacterium) AND (controlled trial) AND (morbidity OR mortality OR complication OR adverse effect OR NICU OR "hospital stay")) OR title((Preterm OR premature) AND (Infection OR sepsis OR septicemia OR bacteremia OR septicaemia OR bacteraemia OR septic shock) AND (Probiotic OR Lactobacilli OR Lactobacillus OR Bifidobacterium) AND (controlled trial) AND (morbidity OR mortality OR complication OR adverse effect OR NICU OR "hospital stay"))
