# Supplementary material for: Probiotics for preventing neonatal sepsis in preterm neonates: a systematic review and meta-analysis for clinical practice
Source: Epidemiol Health. 2025 Sep 3;47:e2025051. doi: 10.4178/epih.e2025051 (PMC12869123; doi:10.4178/epih.e2025051)
Supplement: Supplementary Material 2. — Study characteristics and outcomes measured [file epih-47-e2025051-Supplementary-2.docx]

## Supplementary Material 2. Study characteristics and outcomes measured

| **No** | **Author** | **Country** | **No.** | **Intervention** | **Comparison** | **Treatment duration** | **Characteristics** | **Primary outcomes** | **Secondary outcomes** | **Risk of bias assessment** |
| --- | --- | --- | --- | --- | --- | --- | --- | --- | --- | --- |
| 1 | Agrawal 2018 (12) | Australia | Probiotic: 77 Placebo: 76 | Probiotic supplementation with Bifidobacterium breve (B. breve) m-16v with the dose of  3 x 10^9^ cfu/day, single dose via orogastric feeding tube | Placebo (Maltodextrin) | Until Post-Menstrual Age (PMA) of 37 weeks or discharge | **Gestational age (weeks):** Probiotic: 29 (IQR: 26–30) Placebo: 28 (IQR: 26–29) **Birth weight (grams):** Probiotic: 1090 (IQR: 755–1280) Placebo: 1025 (IQR: 810–1260) **Gender (male):** Probiotic: 45 (58%) Placebo: 41 (54%) **APGAR score <7 at 5 minutes:** Probiotic: 14 (18%) Placebo: 19 (25%) **PDA (Patent ductus arteriosus):** Probiotic: 31 (40%) Placebo: 34 (45%) No significant p-values were reported for these characteristics. | **Neonates with late onset sepsis (n, %):** Probiotic: 17 (22%) Placebo: 12 (16%) | **Length of hospital stay (weeks):** Probiotic: 10 (IQR: 6–14) Placebo: 10 (IQR: 7–14) | Some concerns |
| 2 | Al-Hosni 2011 (13) | United States of America | PS group: 50 C group: 51 | Probiotic supplementation with Lactobacillus rhamnosus GG (500 million CFU) and Bifidobacterium infantis (500 million CFU) added to the first enteral feeding and continued once daily with feedings thereafter | Unsupplemented | Until discharge or 34 weeks postmenstrual age (PMA) | **Gestational age (weeks):** PS group: 25.7 ± 1.4 C group: 25.7 ± 1.4 **Birth weight (grams):** PS group: 778 ± 138 C group: 779 ± 126 **Gender (male):** PS group: 22 (44%) C group: 28 (55%) **APGAR score at 1 min (median):** PS group: 5 C group: 5 **APGAR score at 5 min (median):** PS group: 7 C group: 8 No significant p-values reported for these characteristics. | **Mortality** Probiotic: 3 (6%) Control: 4 (8%) **Any bacterial and fungal sepsis:** Probiotic: 13/50 (26%) Control: 16/51 (31%) | NR | Low |
| 3 | Braga 2011 (14) | Brazil | Probiotics group: 119 Control group: 112 | Oral supplementation with Bifidobacterium breve and Lactobacillus casei in human milk (3.5 × 10^7^ to 3.5× 10^9^ cfu) | Human milk without probiotics | The intervention was initiated on the second day of life and maintained until 30 days of life, diagnosis of NEC, discharge, or death, whichever occurred first. | **Gestational age (weeks):** Probiotics group: 29.5 ± 2.5 Control group: 29.2 ± 2.6 **Birth weight (grams):** Probiotics group: 1194.7 ± 206.3 Control group: 1151.4 ± 224.9 **Gender (male):** Probiotics group: 58 (48.7%) Control group: 55 (49.1%) **APGAR score at 1 min (mean ± SD):** Probiotics group: 5.7 ± 2.4 Control group: 6.1 ± 2.3 **APGAR score at 5 min (mean ± SD):** Probiotics group: 7.9 ± 1.5 Control group: 8.1 ± 1.3 Other characteristics (e.g., use of antenatal corticoid, cesarean delivery) were similar between groups with no significant differences reported. | **Sepsis:** Probiotics group: 40 (33.6%) Control group: 42 (37.5%)  **Death:** Probiotics group: 26 (21.8%) Control group: 27 (24.1%) | NR | Low |
| 4 | Costeloe 2016 (8) | United Kingdom | Probiotic group: 650 Placebo group: 660 | Probiotic supplementation with Bifidobacterium breve BBG-001 (6.7 × 10^7^ to 6.7 × 10^9^ cfu) | Placebo (maize starch in identical powder form) | Administered daily until 36 weeks’ postmenstrual age (PMA) | **Gestational Age at Birth (weeks):** Probiotic Group (n=650): Median: 28.0 weeks Interquartile Range (IQR): 26.1 to 29.4 weeks Placebo Group (n=660): Median: 28.0 weeks IQR: 26.1 to 29.6 weeks **Birth Weight (grams):** Probiotic Group: Mean: 1011 g Standard Deviation (SD): ± 269 g Placebo Group: Mean: 1006 g SD: ± 273 g **Gender (Male):** Probiotic Group: 374 (57.5%) Placebo Group: 370 (56.1%) **Antenatal Steroid Use:** Probiotic Group: Yes, started within 24 hours of birth: 168 (26.1%) Yes, started over 24 hours before birth: 412 (63.9%) Placebo Group: Yes, started within 24 hours of birth: 167 (25.5%) Yes, started over 24 hours before birth: 440 (67.1%) **Mode of Delivery:** Vaginal Delivery: Probiotic Group: 309 (47.5%) Placebo Group: 310 (47.0%) Cesarean Before Labor Onset: Probiotic Group: 221 (34.0%) Placebo Group: 204 (31.0%) Cesarean After Labor Onset: Probiotic Group: 120 (18.5%) Placebo Group: 145 (22.0%) | **Late-Onset Sepsis:** Probiotic Group: 73/650 (11.2%) Placebo Group: 77/660 (11.7%) **Death Before Discharge:** Probiotic Group: 54/650 (8.3%) Placebo Group: 56/660 (8.5%) | **Length of Hospital Stay (days):** Probiotic Group: Median: 68 days IQR: 48 to 98 days Placebo Group: Median: 66 days IQR: 46 to 95 days | Some concerns |
| 5 | Cui 2019 (15) | China | Intervention group: 45 Control group: 48 | Probiotic supplementation with Lactobacillus reuteri DSM 17938 (1 × 10^8^ cfu, 5 drops daily) | Unsupplemented | From the first feeding until discharge from the hospital | **Gestational age (mean ± SD):** Intervention group: 32.85 ± 1.39 weeks Control group: 32.56 ± 1.41 weeks **Birth weight (mean ± SD):** Intervention group: 1682 ± 109.03 g Control group: 1714 ± 127.11 g **Gender (M/F):** Intervention group: 25/20 Control group: 20/28 **5-min Apgar score (mean ± SD):** Intervention group: 9.29 ± 0.84 Control group: 9.29 ± 0.82 | **Sepsis (n, %):** Intervention group: 2/45 (4.44%) Control group: 4/48 (8.33%) p-value: 0.446 (not significant) | **Length of hospital stay (days, mean ± SD):** Intervention group: 20.60 ± 5.36 Control group: 23.75 ± 8.57 p-value: 0.036 (significant) | Some concerns |
| 6 | Demirel 2013 (16) | Turkey | Probiotic group: 135 Placebo group: 136 | Oral supplementation with Saccharomyces boulardii (250 mg/day, 5 billion cfu) | Unsupplemented | From the first feeding until discharge from the hospital | **Gestational age (weeks, mean ± SD):** Study group: 29.4 ± 2.3 Control group: 29.2 ± 2.5 **Birth weight (grams, mean ± SD):** Study group: 1164 ± 261 g Control group: 1131 ± 284 g **Gender (Female, n, %):** Study group: 66 (48.9%) Control group: 70 (51.5%) **Cesarean section (n, %):** Study group: 105 (77.7%) Control group: 113 (83.0%) **Apgar score at 5 minutes (median, min-max):** Study group: 6 (1-7) Control group: 6 (1-7) **Prenatal steroid use (n, %):** Study group: 74 (54.8%) Control group: 72 (52.9%) **Prolonged rupture of amniotic membrane (PROM, n, %):** Study group: 12 (8.9%) Control group: 11 (8.1%) There were no statistically significant differences reported for these baseline characteristics. | **Clinical sepsis (n, %):** Study group: 47 (34.8%) Control group: 65 (47.8%) p-value: 0.030 (significant) **Culture-proven sepsis (n, %):** Study group: 20 (14.9%) Control group: 21 (15.4%) p-value: 0.906 (not significant) **Death (n, %):** Study group: 5 (3.7%) Control group: 5 (3.6%) p-value: 1.000 (not significant) | **Duration of hospitalization (days, median, min-max):** Study group: 47 (6-120) Control group: 43 (4-134) | Low |
| 7 | Dilli 2015 (17) | Turkey | Probiotic group: 100 Placebo group: 100 | Bifidobacterium lactis (5 × 10⁹ cfu) | Placebo (Maltodextrin) | Up to 8 weeks or until discharge or death, whichever came first | **Gestational age (weeks, mean ± SD):** Probiotic: 28.8 ± 1.9 Placebo: 28.2 ± 2.2 **Birth weight (grams, mean ± SD):** Probiotic: 1236 ± 212 Placebo: 1147 ± 271 **Gender (male, n, %):** Probiotic: 53 (53%) Placebo: 58 (58%) **Apgar score at 5 minutes (median, IQR):** Probiotic: 7 (6-8) Placebo: 7 (6-8) **Cesarean delivery (n, %):** Probiotic: 35 (35%) Placebo: 37 (37%) No significant differences in baseline characteristics among the groups. | **Late-onset sepsis, clinical (n, %):** Probiotic: 29 (29%) Placebo: 45 (45%) **Late-onset sepsis, proven (n, %):** Probiotic: 8 (8%) Placebo: 13 (13%) **Mortality (n, %):** Probiotic: 3 (3%) Placebo: 12 (12%) | **NICU stay (days, median, IQR):** Probiotic: 37 (27-50) Placebo: 50 (31-70) | Low |
| 8 | Dongol Singh 2017 (18) | Nepal | Probiotic group: 37 Placebo group: 35 | Probiotic supplementation with Lactobacillus rhamnosus 35 (0.8 mg for infants >1500 gms and 0.4 mg for infants <1500 gms) mixed in 2 ml of expressed breast milk, administered twice daily | Unsupplemented | Until full feeding was reached | **Gestational age (weeks, mean ± SD):** Probiotic group: 32.6 ± 2.2 Placebo group: 32.6 ± 2.2 **Birth weight (groups):** Low birth weight (<2000 gms): Probiotic group: 25 (67.6%) Placebo group: 26 (74.3%) **Very low birth weight (<1500 gms):** Probiotic group: 11 (29.7%) Placebo group: 9 (25.7%) **Extremely very low birth weight (<1000 gms):** Probiotic group: 1 (2.7%) Placebo group: 0 (0%) **Gender (male):** Probiotic group: 16 (43.2%) Placebo group: 16 (45.7%) **Mode of delivery:** Normal vaginal delivery: Probiotic group: 29 (78.4%) Placebo group: 27 (77.1%) Instrumental delivery: Probiotic group: 0 (0.0%) Placebo group: 1 (2.8%) Cesarean section: Probiotic group: 8 (21.6%) Placebo group: 7 (20.0%) Prenatal steroid: Probiotic group: 25 (67.6%) Placebo group: 24 (68.6%) | **Mortality (n, %):** Probiotic group: 3 (8.1%) Placebo group: 3 (8.7%) p-value: 0.63 (not significant) | NR | Some concerns |
| 9 | Dutta 2015 (9) | India | Group A = 38  Group B = 38  Group C = 38  Group D = 35 | Group A (High-dose long course): 10^10^ cells 12-hourly for 21 days Group B (High-dose short course): 10^10^ cells 12-hourly for 14 days followed by placebo from days 15–21 Group C (Low-dose long course): 10^9^ cells 12-hourly for 21 days Probiotic strains used: Mixture of Lactobacillus and Bifidobacterium species | Group D (Placebo): Placebo for 21 days | 21 days | **Gestational age (weeks, mean ± SD):** Group A: 30.64 ± 1.64 Group B: 31.08 ± 1.88 Group C: 30.89 ± 1.96 Group D: 30.82 ± 1.72 **Birth weight (grams, mean ± SD):** Group A: 1286.08 ± 264.76 Group B: 1335.97 ± 284.45 Group C: 1413.32 ± 296.56 Group D: 1252.27 ± 309.31 **Male gender (n, %):** Group A: 25 (65.8%) Group B: 23 (60.5%) Group C: 20 (52.6%) Group D: 23 (65.7%) **Vaginal delivery (n, %):** Group A: 30 (71%) Group B: 24 (52.6%) Group C: 25 (57.9%) Group D: 21 (48.6%) **Apgar score at 5 minutes (median, IQR):** Group A: 9 (8, 9) Group B: 9 (8, 9) Group C: 8.5 (8, 9) Group D: 9 (8, 9) **Antenatal steroids given (n, %):** Group A: 24 (63.2%) Group B: 26 (68.4%) Group C: 22 (57.9%) Group D: 19 (54.3%) | **All sepsis (n):** Group A: 12 Group B: 10 Group C: 12 Group D: 12 **Culture-positive sepsis (n):** Group A: 2 Group B: 0 Group C: 3 Group D: 2 **Death (n, %):** Group A: 3 Group B: 3 Group C: 2 Group D: 2 | NR | Some concerns |
| 10 | Fernández-Carrocera 2013 (19) | Mexico | Probiotic group: 75 Placebo group:  75 | Daily feeding supplementation with a multispecies probiotic containing: Lactobacillus acidophilus Lactobacillus rhamnosus Lactobacillus casei Lactobacillus plantarum Bifidobacterium infantis Streptococcus thermophilus 1 g of probiotic powder per day added to regular feedings | Unsupplemented | Until discharge or until the clinical condition precluded enteral intake | **Gestational age (weeks, median, min–max):** Study group: 31.2 (26–35.4) Control group: 31 (27–36) p-value: 0.351 (not significant) **Birth weight (grams, median, min–max):** Study group: 1090 (580–1495) Control group: 1170 (540–1492) p-value: 0.385 (not significant) **Small for gestational age (n, %):** Study group: 59 (78.7%) Control group: 58 (77.3%) p-value: 0.844 (not significant) **Apgar score at 1 min (n, %):** 0–3: Study group: 9 (12.0%), Control group: 6 (8%) 4–6: Study group: 17 (22.7%), Control group: 23 (30.7%) 7: Study group: 49 (65.3%), Control group: 46 (61.3%) p-value: 0.450 (not significant) **Apgar score at 5 min (n, %):** 0–3: 0 in both groups 4–6: Study group: 5 (6.7%), Control group: 2 (2.7%) ≥7: Study group: 70 (93.3%), Control group: 73 (97.3%) p-value: 0.240 (not significant) **Use of prenatal steroids (n, %):** Study group: 50 (66.7%) Control group: 53 (70.7%) p-value: 0.590 (not significant) **Use of surfactant (n, %):** Study group: 57 (76%) Control group: 52 (69.3%) p-value: 0.360 (not significant) | **Mortality (n, %):** Study group: 1 (1.3%) Control group: 7 (9.3%) RR: 0.14 (95% CI: 0.01 to 1.13) p-value: 0.063 (not significant) **Sepsis (n, %):** Study group: 42 (56%) Control group: 44 (58.7%) p-value: NR (not reported as significant) | NR | Some concerns |
| 11 | Hays 2016 (20) | France | Probiotic group: 147 Placebo group:  52 | Probiotic supplementation with the following subgroups: Group P1: Bifidobacterium lactis Group P2: Bifidobacterium longum Group P3: Combination of Bifidobacterium lactis and Bifidobacterium longum  All at 10^9^ cfu cfu/day | Placebo (maltodextrin) | 4 to 6 weeks depending on the gestational age of the infants: 4 weeks for those with a gestational age (GA) at birth of ≤29 weeks 6 weeks for those with a GA at birth of ≤28 weeks | **Gestational age (weeks, median):** Control group: 29.4 (27.9; 30.6) Probiotic group: 29.0 (28.1; 30.1) p-value: NR **Birth weight (grams, median):** Control group: 1170 (1055; 1370) Probiotic group: 1170 (1000; 1320) p-value: NR **Gender (male, %):** Control group: 67.3% Probiotic group: 45.8% p-value: NR **Cesarean section (%):** Control group: 75% Probiotic group: 79.3% p-value: NR **Surfactant use (%):** Control group: 63.5% Probiotic group: 59.3% p-value: NR | Late-onset sepsis (n, %):  Control group: 19 (9.6%)  Probiotic group: 17 (8.5%)  Mortality (n, %):  Control group: 1 (1.9%)  Probiotic group: 4 (2.7%) | NR | Low |
| 12 | Jacobs 2013 (21) | Australia | 548 in the probiotic group, 551 in the control group | Daily administration of a probiotic combination: Bifidobacterium infantis (BB–02 300 × 10^6^ cfu) Streptococcus thermophilus (TH–4 350 × 10^6^ cfu) Bifidobacterium lactis (BB-12 350 × 10^6^ cfu) Total: 1 × 10^9^ cfu | Placebo (maltodextrin) | Administered until discharge from hospital or term corrected age | **Gestational age (weeks, mean ± SD):** Probiotic group: 27.9 ± 2.0 Control group: 27.8 ± 2.0 **Birth weight (grams, mean ± SD):** Probiotic group: 1063 ± 259 Control group: 1048 ± 260 **Gender (male, %):** Probiotic group: 49.6% Control group: 54.4% **5-minute Apgar score (median [IQR]):** Probiotic group: 8 [7–9] Control group: 8 [7–9] **Cesarean delivery (n, %):** Probiotic group: 65.5% Control group: 68.4% | **Definite late-onset sepsis (n, %):** Probiotic group: 72 (13.1%) Control group: 89 (16.2%) p-value: 0.16 (not significant) **Clinical late-onset sepsis (n, %):** Probiotic group: 75 (13.7%) Control group: 83 (15.1%) p-value: 0.52 (not significant) **Composite outcome of late-onset sepsis (definite or clinical) (n, %)**: Probiotic group: 129 (23.5%) Control group: 146 (26.5%) p-value: 0.26 (not significant) **Mortality (n, %):** Probiotic group: 27 (4.9%) Control group: 28 (5.1%) p-value: 0.91 (not significant) | **Length of primary hospital admission (days, median [IQR]):** Probiotic group: 71 [54–92] Control group: 74 [58–93] | Low |
| 13 | Kaban 2019 (22) | Indonesia | 47 in the probiotic group and 47 in the placebo group | Oral administration of Lactobacillus reuteri DSM 17938 (five drops per day, equivalent to 10^8^ CFU/day | Placebo (mixture of pharmaceutical-grade medium-chain triglycerides and sunflower oil with pharmaceutical-grade silicon dioxide) | Intervention was given for at least 7 days or until the subject was discharged, experienced NEC, or died. | **Gestational age (weeks, mean [range]):** Probiotic group: 33 (28–34) Placebo group: 33 (28–34) **Birth weight (grams, mean [range]):** Probiotic group: 1,520 (1,035–1,800) Placebo group: 1,605 (1,060–1,800) **Gender (male, %):** Probiotic group: 59.6% Placebo group: 36.2% p-value: 0.02 (significant) **Cesarean section (n, %):** Probiotic group: 83% Placebo group: 85.1% p-value: 0.78 (not significant) **APGAR score at 1 minute (median [range]):** Probiotic group: 7 (5–9) Placebo group: 7 (3–9) **APGAR score at 5 minutes (median [range]):** Probiotic group: 9 (6–10) Placebo group: 9 (5–10) **Steroid administration (n, %):** Probiotic group: 74.5% Placebo group: 74.5% p-value: 1.00 (not significant) | **Proven sepsis (n, %):** Probiotic group: 1 (2.1%) Placebo group: 3 (6.4%) p-value: 0.62 (not significant) **Mortality (n, %):** Probiotic group: 1 (2.1%) Placebo group: 4 (8.5%) p-value: 0.36 (not significant) | **Length of stay (days, median [range]):** Probiotic group: 27 (8–72) Placebo group: 27 (11–73) p-value: 0.28 (not significant) | Low |
| 14 | Kanic 2019 (23) | Slovenia | 80 very low birth weight (VLBW) infants (40 in the probiotic group and 40 in the control group) | A combination of probiotics: Lactobacillus acidophilus (subsp. L. gasseri) Bifidobacterium infantis Enterococcus faecium Administered at a dose of 1.2 × 10^7^ cfu twice daily until discharge. | Unsupplemented | Probiotic administration began with the first portions of milk and continued until discharge. | **Gestational age (weeks, IQR):** Probiotic group: 28.0 (27.0–30.0) Control group: 29.0 (26.2–30.0) p-value: Not significant (Ns) **Birth weight (grams, mean ± SD):** Probiotic group: 1104.1 ± 233.2 Control group: 1024.3 ± 249.9 p-value: Ns **Gender (male, %):** Probiotic group: 55.0% Control group: 67.5% p-value: Ns **APGAR score at 5 minutes (IQR):** Probiotic group: 8.0 (7.0–8.0) Control group: 7.0 (6.0–8.0) p-value: Ns | **Late-onset sepsis (n, %):** Probiotic group: 16 (40.0%) Control group: 29 (72.5%) p-value: 0.006 (significant) **Mortality (n, %):** Probiotic group: 2 (5.0%) Control group: 3 (7.5%) p-value: Ns | **Postmenstrual age at discharge (weeks, IQR):** Probiotic group: 37.0 (36.0–39.0) Control group: 38.0 (37.0–40.0) p-value: 0.02 (significant) | Some concerns |
| 15 | Mihatsch 2010 (24) | Germany | 183 very low birth weight (VLBW) infants (93 in the Bifidobacterium lactis group and 90 in the placebo group) | Bifidobacterium lactis BB12 suspension at a dosage of 6 × 10^9^ CFU/kg/day | Placebo (human milk fortifier powder only) | Intervention was given from initiation of milk feedings until the 42nd day of life. | **Gestational age (weeks, mean ± SD):** B. lactis group: 26.6 ± 1.8 Placebo group: 26.7 ± 1.7 **Birth weight (grams, mean ± SD):** B. lactis group: 856 ± 251 Placebo group: 871 ± 287 **Gender (male, %):** B. lactis group: 60.4% Placebo group: 52.8% **APGAR score at 5 minutes (mean ± SD):** B. lactis group: 7.9 ± 1.8 Placebo group: 7.8 ± 1.4 **Small for gestational age (SGA, %):** B. lactis group: 7.7% Placebo group: 14.6% | **Positive blood culture (n, %):** B. lactis group: 28 (20%) Placebo group: 29 (16%) **Mortality (n, %):** B. lactis group: 2 (2%) Placebo group: 1 (1%) p-value: Not significant | NR | Low |
| 16 | Niekerk 2015 (25) | South Africa | 184 premature infants (74 HIV-exposed and 110 HIV-unexposed) | Probiotic supplementation consisting of Lactobacillus rhamnosus GG and Bifidobacterium infantis (1 × 10^9^ CFU/day) | Placebo | Probiotics or placebo were administered daily until discharge or death. | HIV-exposed infants: Probiotic group: 38 Placebo group: 36 HIV-unexposed infants: Probiotic group: 55 Placebo group: 55 | **Positive blood culture (n, %):** Probiotic group: 5 (14%) Placebo group: 3 (8%) p-value: 1.00 (not significant) **Mortality (n, %):** Probiotic group: 4 (5.4%) Placebo group: 7 (6%) p-value: 0.79 (not significant) | NR | Low |
| 17 | Oncel 2014 (26) | Turkey | 400 very low birth weight (VLBW) infants (200 in the probiotic group and 200 in the placebo group) | Oral administration of Lactobacillus reuteri DSM 17938 (1 × 10^8^ CFU/day) | Placebo (oil-based suspension without active probiotics) | Intervention continued until discharge or death. | **Gestational age (weeks, mean ± SD):** Probiotic group: 28.2 ± 2.4 Placebo group: 27.9 ± 2.5 **Birth weight (grams, mean ± SD):** Probiotic group: 1071 ± 274 Placebo group: 1048 ± 298 **Gender (male, %):** Probiotic group: 54% Placebo group: 49% **APGAR score at 5 minutes (median [range]):** Probiotic group: 8 (5–9) Placebo group: 8 (5–9) | **Proven sepsis (n, %):** Probiotic group: 13 (6.5%) Placebo group: 25 (12.5%) p-value: 0.041 (significant) | **Duration of hospitalization (days, median [range]):** Probiotic group: 38 (10–131) Placebo group: 46 (10–180) p-value: 0.022 (significant) | Low |
| 18 | Patole 2014 (27) | Australia | 159 preterm neonates (Probiotic: 79, Placebo: 80) | Bifidobacterium breve M-16V supplementation (3 × 10^9^ CFU/day) | Placebo group received dextrin as a control | The intervention continued until the corrected age of 37 weeks | **Gestational age (weeks, median [range]):** Probiotic: 29 (26–30; 23–32) Placebo: 28 (26–29; 23–33) **Birth weight (grams, median [range]):** Probiotic: 1090 (755–1280; 466–1830) Placebo: 1025 (810–1260; 480–1770) **Gender (male, %):** Probiotic: 58% Placebo: 54% **APGAR score <7 at 5 minutes (n, %):** Probiotic: 14 (18%) Placebo: 19 (25%) | **Late onset sepsis (n, %):** Probiotic: 17 (22%) Placebo: 12 (16%) p-value: 0.410 (not significant) **All cause deaths (n, %):** Both groups: 0 (0%) | **Length of hospital stay (weeks, median [range]):** Probiotic: 10 (6–14; 2–61) Placebo: 10 (7–14; 3–60) p-value: 0.812 (not significant) | Low |
| 19 | Rasania 2023 (28) | India | Total: 123 preterm neonates Group A: 41 neonates Group B: 42 neonates Group C: 40 neonates | Group A: Probiotic sachet containing lyophilized Lactobacillus rhamnosus GG (6 billion CFUs daily) Group B: Probiotic drops containing lyophilized Lactobacillus rhamnosus GG (2 billion CFUs daily) | Group C: No probiotic (Control group) | Probiotic was administered until the neonate reached the corrected gestational age of 36 weeks or a maximum of 4 weeks. | **Gestational Age (Mean ± SD, Weeks):** Group A: 32.20 ± 1.82 Group B: 32.29 ± 1.60 Group C: 31.78 ± 1.75 **Birth Weight (Mean ± SD, Grams):** Group A: 1578.78 ± 302.13 Group B: 1549.45 ± 286.89 Group C: 1435.75 ± 344.02 **Gender (Male, %):** Group A: 60.98% Group B: 42.86% Group C: 60.00% | **Late-onset sepsis (LOS) (n, %):** Group A: 5 (12.20%) Group B: 3 (7.14%) Group C: 3 (7.50%) p-value: 0.7 (not significant) **Death (n, %):** Group A: 0 (0%) Group B: 1 (2.38%) Group C: 1 (2.50%) p-value: NR | **Length of hospital stay (days, Mean ± SD):** Group A: 14.11 ± 10.95 Group B: 16.1 ± 10.67 Group C: 20.38 ± 14.6 p-value: 0.04 (significant between Group A and Group C) | Some concerns |
| 20 | Rojas 2012 (29) | United States of America | 750 infants were enrolled (372 in the probiotic group and 378 in the placebo group) | Prophylactic administration of Lactobacillus reuteri DSM 17938 (5 drops of an oil-based suspension containing 10^8^ cfu) | Placebo (an identical vial containing only the oil base) | Daily administration from within the first 48 hours after birth until death or discharge from the hospital | **Number of participants:** Probiotic: 372 Placebo: 378 **Gender (male):** Probiotic: 186 (50%) Placebo: 185 (49%) **Gestational age (weeks, median):** Probiotic: 32 (IQR 30-33) Placebo: 32 (IQR 29-33) **Birth weight (g, median):** Probiotic: 1530 (IQR 1253-1750) Placebo: 1516 (IQR 1129-1750) **APGAR score at 5 minutes (median):** Probiotic: 9 (IQR 8-9) Placebo: 9 (IQR 8-9) | **Positive culture:** Probiotic: 34 (9.1%) Placebo: 40 (10.6%) **Death:** Probiotic: 22 (5.9%) Placebo: 28 (7.4%) | **Duration of hospitalization (days, median):** Probiotic: 20 (IQR 11-33) Placebo: 20 (IQR 11-38) P = .53 | Low |
| 21 | Romeo 2011 (30) | Italy | Total: 249 preterm infants Group I (Lactobacillus reuteri): 83 Group II (Lactobacillus rhamnosus): 83 Group III (Control): 83 | Group I: Supplementation with Lactobacillus reuteri (ATCC 55730), 5 drops daily Group II: Supplementation with Lactobacillus rhamnosus (ATCC 53103), 1 capsule daily | Unsupplemented | Probiotics were administered from the first 72 hours after hospitalization for 6 weeks or until discharge from the NICU | **Gestational Age (weeks, mean ± SD):** Group I (Lactobacillus reuteri): 33.8 ± 1.8 Group II (Lactobacillus rhamnosus): 33.3 ± 1.6 Control: 33.3 ± 2.1 **Birth Weight (grams, mean ± SD):** Group I (Lactobacillus reuteri): 1998.7 ± 439 Group II (Lactobacillus rhamnosus): 1940.7 ± 590 Control: 1945.7 ± 465 **Gender (Male/Female):** Group I (Lactobacillus reuteri): 44/39 Group II (Lactobacillus rhamnosus): 45/38 Control: 46/37 | **Bacterial Infections (n):** Group I (Lactobacillus reuteri): 0 Group II (Lactobacillus rhamnosus): 1 Control: 5 | **Days of Hospitalization (mean ± SD):** Group I (Lactobacillus reuteri): 17.8 ± 7.9 Group II (Lactobacillus rhamnosus): 26.9 ± 15.7 Control: 31.3 ± 16.3 p-value: Significant (p < 0.0001) between L. reuteri and the other groups | Low |
| 22 | Roy 2014 (31) | India | Total: 112 preterm neonates Group I (Probiotics): 56 Group II (Placebo): 56 | Probiotic supplementation with Lactobacillus acidophilus (1.25 billion CFU), Bifidobacterium longum (0.125 billion CFU), Bifidobacterium bifidum (0.125 billion CFU), and Bifidobacterium lactis (1.0 billion CFU) per 1 g sachet. | Control group received sterile water as placebo | Probiotics were administered daily from the first 72 hours for 6 weeks or until discharge from the NICU | **Gestational Age (mean ± SD):** Probiotic group: 32 ± 2 weeks Control group: 32.2 ± 2 weeks p-value: 0.921 (not significant) **Birth Weight (mean ± SD):** Probiotic group: 1192 ± 341 g Control group: 1069 ± 365 g p-value: NR **Gender (Male/Female):** Probiotic group: 14/42 Control group: 16/40 p-value: 0.789 (not significant) Other characteristics (e.g., small for gestational age, socioeconomic status, maternal diabetes, etc.) were balanced between groups with no significant differences. | **Late-onset sepsis incidence (%):** Probiotic group: 55.4% (31/56) Control group: 75% (42/56) p-value: 0.02 (significant) **Death (%):** Probiotic group: 7 (15.2%) Control group: 8 (17.4%) p-value: 0.5 (not significant) | **Duration of hospitalization (mean ± SD):** Probiotic group: 25.77 ± 9.16 days Control group: 31.21 ± 12.67 days p-value: 0.002 (significant) | High |
| 23 | Saengtawesin 2014 (32) | Thailand | Total: 60 preterm infants Study Group: 31 Control Group: 29 | Oral probiotic supplementation with Infloran® (contains Lactobacillus acidophilus 1x10^9^ and Bifidobacterium bifidum 1x10^9^ organisms), dose of 125 mg/kg/dose twice a day. | Control group received breast milk or premature formula alone, with no probiotic supplementation. | From the start of feeding until 6 weeks or discharge. | Gestational Age (weeks, mean ± SD): Study Group: 31.0 ± 1.82 Control Group: 30.59 ± 1.76 p-value: 0.34 Birth Weight (grams, mean ± SD): Study Group: 1,250.1 ± 179.26 Control Group: 1,207.72 ± 199.35 p-value: 0.49 Gender (Male/Female): Study Group: 19/12 Control Group: 11/18 p-value: 0.03 (significant) Other characteristics (e.g., PROM, preeclampsia, prenatal steroid treatment, use of surfactant, antibiotic use) were balanced between groups, with no significant differences except for antibiotic use at 2 weeks, which was higher in the control group (p = 0.03). | **Death (%):** Study Group: 0% Control Group: 0% **Late-onset sepsis:** Study Group: 6.45% (2/31), Control Group: 3.44% (1/29) p-value: 0.53 (not significant) | NR | Some concerns |
| 24 | Sari 2011 (33) | Turkey | Total: 221 infants Study Group: 110 Control Group: 111 | Oral probiotic supplementation with Lactobacillus sporogenes at a dose of 350 million CFU added to breast milk or formula, given once a day. | Control group received breast milk or formula without the addition of Lactobacillus sporogenes. | From the start of feeding until discharge from the hospital. | Gestational Age (weeks, mean ± SD): Study Group: 29.5 ± 2.4 Control Group: 29.7 ± 2.4 Birth Weight (grams, mean ± SD): Study Group: 1231 ± 262 Control Group: 1278 ± 282 Gender (Male/Female): Study Group: 60/50 Control Group: 62/49 P-values: All comparisons between groups for baseline characteristics were not statistically significant. | **Death or NEC (%):** Study Group: 8.2% (9/110) Control Group: 11.7% (13/111) p-value: 0.515 (not significant) **Death attributable to NEC (%):** Study Group: 0% (0/110) Control Group: 0.9% (1/111) p-value: 1.000 (not significant) **Sepsis (culture-proven) (%):** Study Group: 26.4% (29/110) Control Group: 23.4% (26/111) p-value: 0.613 (not significant) | NR | Low |
| 25 | Serce 2013 (34) | Turkey | 208 (104 in the intervention group, 104 in the control group) | Saccharomyces boulardii (50 mg/kg every 12 hours) | Placebo (distilled water, 1 mL per dose) | From the first feed until discharge (Median duration: 44 days) | Number of participants: Intervention: 104 Control: 104 Birth weight (g, mean ± SD): Intervention: 1126 ± 232 Control: 1162 ± 216 Gestational age (weeks, mean ± SD): Intervention: 28.8 ± 2.2 Control: 28.7 ± 2.1 Gender (male, n, %) Intervention: 51 (49%) Control: 56 (53.8%) APGAR score (5th min, median): Intervention: 8 (6–8) Control: 7 (6–8) Statistical significance: No significant differences in baseline characteristics between groups. | **Late onset culture-proven sepsis:** Intervention: 19 (18.3%) Control: 25 (24.3%) Not significant (p = 0.29) **Deaths (all causes):** Intervention: 5 (4.8%) Control: 4 (3.8%) Not significant (p = 0.74) | **Duration of hospitalization (median, 25%-75%):** Intervention: 39 days (28–60) Control: 43 days (29–60) Not significant (p = 0.62) | Low |
| 26 | Shadkam 2015 (35) | Iran | Intervention: 30 Placebo: 30 | Oral administration of probiotics containing Lactobacillus reuteri (DSM 17938) 1 drop/kg | Placebo group (0.5 ml distilled water) | Until full enteral feeding was reached, which was defined as a volume of 120 ml/kg per day. | Age (gestational age): Intervention: 30.87 ± 1.90 weeks Placebo: 30.97 ± 1.94 weeks Gender: Intervention: 46.7% male Placebo: 53.3% male Weight: Intervention: 1396.33 ± 234.55 grams Placebo: 1418.67 ± 328.47 grams | **Sepsis**: Intervention: 4 (13.3%) Placebo: 10 (33.4%) P-value: 0.109 **Mortality:** Intervention: 1 (3.3%) Placebo: 2 (6.7%) P-value: 0.5 | NR | Low |
| 27 | Sowden 2022 (36) | South Africa | 200 (100 in the probiotic group, 100 in the placebo group) | Multi-strain probiotic formulation (Labinic™), containing Lactobacillus acidophilus (0.67 billion CFUs), Bifidobacterium bifidum (0.67 billion CFUs), and Bifidobacterium infantis (0.67 billion CFUs) | Placebo | Up to 28 days | Number of participants: Probiotic (100), Placebo (100) Gestational age: Probiotic (mean 29 weeks), Placebo (mean 30 weeks) Gender: Probiotic (47% male, 53% female), Placebo (37% male, 63% female) Weight: Probiotic (mean 1,174 g), Placebo (mean 1,150 g) APGAR score: Probiotic >7 (89%), Placebo >7 (89%) HIV exposure: Probiotic (22%), Placebo (26%) | **Neonates with positive blood culture (n, %):** Probiotic: 2 (2%) Placebo: 13 (13%) **Death from sepsis (n, %):** Probiotic: 2 (2%) Placebo: 3 (3%) | **Length of NICU Stay:** Mean of 21.35 days for probiotic group and 21.70 days for placebo group | Low |
| 28 | Strus 2018 (37) | Poland | 181 neonates (90 in the probiotic group, 91 in the placebo group) | Oral probiotic supplementation (Lactobacillus rhamnosus KL53A and Bifidobacterium breve PB04) of 10^6^ cfu | Placebo | 6 weeks | Number of participants: Probiotic group: 90, Placebo group: 91 Gestational age (weeks): Probiotic group: 29.73 (±2.26), Placebo group: 29.67 (±2.32), p=0.9457 Birth weight (grams): Probiotic group: 1281.24 (±281.18), Placebo group: 1350.11 (±292.18), p=0.1175 APGAR score: Probiotic group: 4 (1.14%) - 10 (9.09%), Placebo group: 4 (1.14%) - 10 (14.77%) | **Late-Onset Sepsis (LOS):** Probiotic group: 11 cases (12.2%) Placebo group: 7 cases (7.7%) p-value: NR **Sepsis caused by Staphylococcus spp.:** Probiotic group: 2 cases (2.2%) Placebo group: 4 cases (4.4%) p-value: NR **All-Cause Mortality:** Probiotic group: 2 deaths (2.2%) Placebo group: 4 deaths (4.4%) p-value: NR | NR | Low |
| 29 | Tewari 2015 (38) | India | Extreme preterm: Placebo: 59, Probiotic: 61 Very preterm: Placebo: 62, Probiotic: 62 | Probiotic supplementation with Bacillus clausii (2.4 x 10^9^ spores per day) | Placebo (sterile water) | 6 weeks or until discharge, death, or occurrence of sepsis, whichever was earlier. | Age (Gestational Age): Extreme preterm: 27-28 weeks (Placebo: 45%, Probiotic: 46%); 29-30 weeks (Placebo: 55%, Probiotic: 54%) Very preterm: 31-32 weeks (Placebo: 69%, Probiotic: 68%); 33 weeks (Placebo: 31%, Probiotic: 32%) Gender: Extreme preterm: Female (Placebo: 47%, Probiotic: 48%) Very preterm: Female (Placebo: 48%, Probiotic: 53%) Birth weight (mean ± SD): Extreme preterm: Placebo: 713g ± 36g, Probiotic: 715g ± 29g Very preterm: Placebo: 1260g ± 282g, Probiotic: 1280g ± 208g | **Incidence of definite and probable sepsis:** Extreme preterm: Placebo: 29% (17/59), Probiotic: 23% (14/61) Very preterm: Placebo: 13% (8/62), Probiotic: 10% (6/62) **Mortality (all causes):** Extreme preterm: Placebo: 15% (9/59), Probiotic: 13% (8/61) Very preterm: Placebo: 8% (5/62), Probiotic: 7% (4/62) | NR | Some concerns |
| 30 | Totsu 2023 (39) | Japan | 283 very low birth weight (VLBW) infants: 153 in the intervention group (B group) and 130 in the placebo group (P group). | Administration of Bifidobacterium bifidum OLB6378 (approximately 2.5 × 10^9^ viable cells/500 mg) suspended in 0.5 mL warm water, breast milk, or infant formula, given enterally within 48 hours after birth, then twice daily until the infant’s body weight reached 2000 g | Placebo group (500 mg dextrin given in the same manner as the probiotic). | Until the infant’s body weight reached 2000 g. | Number of Participants: 153 (Intervention) vs. 130 (Placebo) Gestational Age (weeks, mean ± SD): 28.6 ± 2.9 (Intervention) vs. 28.5 ± 3.3 (Placebo) Birth Weight (g, mean ± SD): 1016 ± 289 (Intervention) vs. 998 ± 281 (Placebo) Multiple Birth: 29 (19.0%) (Intervention) vs. 27 (20.8%) (Placebo) Antenatal Steroid Use: 101 (66.0%) (Intervention) vs. 66 (50.8%) (Placebo), p < 0.05 Cesarean Section: 91 (59.5%) (Intervention) vs. 103 (79.2%) (Placebo), p < 0.05 Male: 87 (56.9%) (Intervention) vs. 71 (54.6%) (Placebo) Apgar Score at 1 min: Median (25th, 75th %) 6 (4, 8) (Intervention) vs. 5 (3, 7) (Placebo) Apgar Score at 5 min: Median (25th, 75th %) 8 (7, 9) (Intervention) vs. 7 (6, 9) (Placebo) | Sepsis: 13 (8.5%) (Intervention) vs. 17 (13.1%) (Placebo) - Not significant.  Sepsis at or later than 7 days after birth: 6 (3.9%) (Intervention) vs. 10 (10.0%) (Placebo) - Significant, p < 0.05  Mortality: 2 deaths in the intervention group vs. 0 in the placebo group (not significant). | **Length of Hospital Stay:** 92.3 ± 44.5 days (Intervention) vs. 92.9 ± 40.2 days (Placebo) - Not significant. | High |
| 31 | Xu 2016 (40) | China | 125 neonates were enrolled; 63 in the treatment group and 62 in the control group | Probiotic treatment with Saccharomyces boulardii CNCM I-745 at a dosage of 50 mg/kg twice daily. | Control group received no probiotics. | The study period lasted until the 28th day after birth or until the infant was discharged from the hospital, with a minimum intervention duration of at least 7 days. | Number of participants (intervention vs. comparison): 51 vs. 49 (after dropouts) Birthweight (g): 1947 ± 54 (intervention) vs. 1957 ± 51 (control), p > 0.05 Gestational age (weeks): 33.0 ± 0.72 (intervention) vs. 33.0 ± 1.04 (control), p > 0.05 Boys/girls: 27/24 (intervention) vs. 24/25 (control), p > 0.05 Respiratory difficulties: 5 (intervention) vs. 6 (control), p > 0.05 Hyperbilirubinemia: 16 (31.4%) (intervention) vs. 14 (28%) (control), p > 0.05 Anemia: 23 (45.1%) (intervention) vs. 25 (51.0%) (control), p > 0.05 Antibiotic treatment: 11 (21.6%) (intervention) vs. 9 (18.4%) (control), p > 0.05 | **Incidence of sepsis:** 4 (7.8%) (intervention) vs. 6 (12.2%) (control), p > 0.05 | **Hospital stay (days):** 23.3 ± 1.6 (intervention) vs. 28.0 ± 1.8 (control), p = 0.035 | Some concerns |
