# Supplementary material for: Probiotics for preventing neonatal sepsis in preterm neonates: a systematic review and meta-analysis for clinical practice
Source: Epidemiol Health. 2025 Sep 3;47:e2025051. doi: 10.4178/epih.e2025051 (PMC12869123; doi:10.4178/epih.e2025051)
Supplement: Supplementary Material 3. — The Revised Cochrane Risk of Bias Tool (RoB 2.0) assessment [file epih-47-e2025051-Supplementary-3.docx]

## Supplementary Material 3. The Revised Cochrane Risk of Bias Tool (RoB 2.0) assessment

| **No.** | **Author** | **Randomization** | **Rationale** | **Deviations from Intended Intervention** | **Rationale** | **Missing Outcome Data** | **Rationale** | **Measurement of the Outcome** | **Rationale** | **Selective Reporting** | **Rationale** |
| --- | --- | --- | --- | --- | --- | --- | --- | --- | --- | --- | --- |
| 1 | Agrawal 2018 (12) | Low | The study clearly describes the randomization process, with the infants being randomized to either the probiotic or placebo group. The baseline characteristics between the two groups were comparable, suggesting that the randomization process was effective in evenly distributing potential confounding factors across the groups. | Some concerns | While the study was double-blinded, there are concerns about potential cross-contamination, as the study mentions the possibility of cross-colonization of the probiotic strain in the placebo group. | Low | The study reports the number of infants included in the analysis, with only a small number of exclusions due to lack of CRP measurements or withdrawal from the study. | Low | The outcome measure is objectively measured using standard methods. There is no indication that the outcome assessors were aware of group assignments, which reduces the risk of bias in outcome measurement. | Low | The study appears to have reported all pre-specified outcomes, and there is no indication of selective reporting. Primary and secondary outcomes are reported |
| 2 | Al-Hosni 2011 (13) | Low | The study was described as a double-blinded, randomized controlled trial. Randomization was performed appropriately, and the allocation to groups was concealed, minimizing the potential for selection bias. No issues were reported regarding baseline differences between groups | Low | The study was double-blinded, with both caregivers and outcome assessors blinded to the intervention. This design minimizes the risk of bias due to deviations from the intended intervention. | Low | The study reports that the analysis was conducted on an intention-to-treat basis. The proportion of missing data was low, and reasons for missing data were provided | Low | The outcome measure is objectively measured using standard methods. There is no indication that the outcome assessors were aware of group assignments, which reduces the risk of bias in outcome measurement. | Low | The study appears to have reported all pre-specified outcomes, and there is no indication of selective reporting. Primary and secondary outcomes are reported |
| 3 | Braga 2011 (14) | Low | The study mentions that randomization was carried out in blocks of 10 using a list of random numbers generated by the subprogram Epitable from Epi-Info 6.04 (Centers for Disease Control and Prevention, Atlanta, GA). Two trained external personnel from the NICU were responsible for randomization, ensuring that the process was concealed | Low | The intervention was initiated on the second day of life for all infants and was maintained until 30 days of life, discharge, diagnosis of NEC, or death. The trial was double-blind | Low | There is no indication of differential loss to follow-up between the groups that would suggest a bias due to missing outcome data | Low | The primary outcome, the occurrence of NEC (Bell’s stage ≥2), was measured using well-established criteria (Bell’s criteria modified by Walsh and Kliegman). The study also maintained blinding of the outcome assessors | Low | The study protocol was registered before the trial (as noted by the trial registration number), and the primary outcome was clearly defined as the occurrence of NEC stage ≥2. The results were reported as planned |
| 4 | Costeloe 2016 (8) | Low | The study describes an adequate method of randomization, where participants were assigned to groups using a computer-generated randomization sequence. Additionally, allocation concealment was ensured by using sequentially numbered, opaque, sealed envelopes. | Some concerns | The study was open-label, meaning neither participants nor clinicians were blinded to the treatment groups. While the study protocol was followed closely, the lack of blinding could have led to deviations from intended interventions that were not systematically reported. | Low | The study reports a low rate of missing outcome data, and it is mentioned that analyses were performed on an intention-to-treat basis | Low | The outcomes were measured using standard and validated methods. Additionally, outcome assessors were blinded to the group assignments | Low | The study protocol was registered, and the outcomes reported in the final publication are consistent with the prespecified outcomes |
| 5 | Cui 2019 (15) | Low | The study uses a random computer-determined allocation method to assign participants, considering gestational age. The study mentions no significant differences in baseline characteristics between groups | Low | The study was double-blinded. The protocol for administering the intervention and control (placebo) was clearly described and consistently followed. | Low | The study accounted for all participants initially randomized, with reasons provided for exclusions. The percentage of exclusions is low, and the reasons for missing data are unlikely to be related to the true outcome | Low | The outcomes were clearly defined, and the measurement methods were standardized and objective | Some concerns | The study reports results for several outcomes, but it is not clear if all pre-specified outcomes were reported. There is some risk that selective reporting may have occurred |
| 6 | Demirel 2013 (16) | Low | The randomization method was well-documented, and the allocation process appears to be free from significant issues that could introduce bias. | Low | The study was blinded, with only the breast milk team aware of group assignments, minimizing the possibility of deviations from the intended interventions | Low | The study had minimal dropouts, and reasons for exclusions were well-documented. There is no indication that the missing data would have led to biased results. | Low | Outcome assessment was likely blinded, as the study indicated that the attending neonatologists evaluating NEC cases were unaware of group assignments | Low | The study reported all primary and secondary outcomes specified in the methods section, with no evidence of selective outcome reporting. The study's registration and adherence to the reporting of registered outcomes supports this judgment. |
| 7 | Dilli 2015 (17) | Low | The study was a multicenter, double-blind, randomized controlled trial. The randomization was done using balanced blocks and sealed envelopes, which were opened only after the infant met the inclusion criteria (feeding enterally and surviving beyond the seventh day). This approach likely ensured adequate randomization and concealment. | Low | The study was double-blind, with both participants and personnel unaware of the group assignments. Additionally, the intervention and placebo products were similar in appearance and preparation | Low | The study followed an intention-to-treat approach and included all eligible infants who survived beyond the seventh day. The report does not indicate differential loss to follow-up across the groups. | Low | The primary and secondary outcomes were clearly defined, and standardized definitions and classifications (e.g., Bell's staging for NEC, Score for Neonatal Acute Physiology Perinatal Extension-II) were used. The outcomes were likely measured similarly across all groups. | Low | The study reported on all prespecified primary and secondary outcomes |
| 8 | Dongol Singh 2017 (18) | Low | The study conducted a randomized, double-blind, placebo-controlled trial with clear randomization procedures. The allocation to the probiotic and placebo groups was done using random selection by lottery | Low | The study maintained blinding and controlled for deviations from the intended intervention. The interventions were administered by nursing staff under the instruction of the researchers | Low | The study reported the number of participants initially enrolled, excluded, and those who completed the study. There was no significant difference in missing data between the groups, and the study included all available data for analysis, | Some concerns | While the study used well-established criteria (Modified Bell’s staging) for diagnosing necrotizing enterocolitis (NEC), there is some concern about potential bias in outcome assessment, as the study did not specify whether outcome assessors were blinded to group allocation | Low | The study reported all pre-specified outcomes, including primary and secondary endpoints. |
| 9 | Dutta 2015 (9) | Low | The randomization was conducted using a block randomization sequence generated online, ensuring that the allocation ratio was 1:1:1:1. Containers were serially numbered and distributed according to the random sequence, which supports a low risk of bias. There was also no significant difference in baseline characteristics among the groups | Some concerns | The study notes that contamination of the placebo group with live probiotic bacteria introduced into the neonatal intensive care unit may have occurred, potentially affecting the outcomes. | Low | The proportion of subjects with unavailable stool samples was not significantly different between groups. Missing data were due to death, leaving against medical advice, or non-passage of stools, which were well-documented and appear unlikely to bias the results significantly​ | Low | The outcomes were clearly defined, and the measurement methods were standardized and objective | Low | The study reports all planned outcomes, including primary and secondary endpoints, and provides detailed results for both, reducing the likelihood of selective reporting |
| 10 | Fernández-Carrocera 2013 (19) | Some concerns | The study states, "Infants were prospectively and randomly assigned to one of two groups using a random digit table, which was handled by the Human Milk Bank staff that was not involved in the care of the patients." While this suggests an effort to minimize bias, the lack of detail on how allocation concealment was maintained introduces some concerns about potential bias. | Low | The study likely has a low risk of bias in this domain. The clinicians and nurses were blinded to the group assignments | Low | The study does not report significant issues with missing data and analyzes the outcomes for all participants who were randomized, | Low | The outcomes were clearly defined, and the measurement methods were standardized and objective | Low | The study reports all planned outcomes, including primary and secondary endpoints, and provides detailed results for both, reducing the likelihood of selective reporting |
| 11 | Hays 2016 (20) | Low | The study employed a pre-established randomization list stratified by the investigating center and gestational age at birth, with a block size of four. This randomization process, along with the use of consecutively numbered, sealed, opaque envelopes, minimizes the risk of bias in group allocation. | Low | The trial was double-blind, with both participants and personnel involved in care and assessment being blinded to group assignments. The intervention and placebo were similar in appearance, reducing the risk of deviations from the intended interventions. | Low | The study reports that 20.3% of subjects discontinued the study prematurely, which is relatively balanced between the intervention and control groups. The reasons for discontinuation are provided and seem unrelated to the intervention | Low | The outcomes were clearly defined, and the measurement methods were standardized and objective | Low | The study reports all planned outcomes, including primary and secondary endpoints, and provides detailed results for both, reducing the likelihood of selective reporting |
| 12 | Jacobs 2013 (21) | Low | Randomization was conducted by an independent unit using statistical software (Stata 9.0), with stratification by center and random block sizes of 2, 4, and 6. This suggests that allocation was adequately concealed, and baseline characteristics between the groups were well balanced | Low | The study was double-blinded, and all staff, parents, and investigators were blinded to the group allocation. The probiotic and placebo were similar in appearance | Low | The study reports outcomes for all randomized participants, with minimal missing data. | Low | The outcomes were clearly defined, and the measurement methods were standardized and objective | Low | The study reports all planned outcomes, including primary and secondary endpoints, and provides detailed results for both, reducing the likelihood of selective reporting |
| 13 | Kaban 2019 (22) | Low | The study used a third-party simple alternating randomization technique, which is a method that reduces selection bias. The trial reported no significant differences between groups in baseline characteristics | Low | The study was double-blinded, and all staff, parents, and investigators were blinded to the group allocation. The probiotic and placebo were similar in appearance | Low | The study reported that no subjects dropped out after allocation to the intervention groups, and all participants were included in the analysis (intention-to-treat analysis). | Low | The outcomes were clearly defined, and the measurement methods were standardized and objective | Low | The study reports all planned outcomes, including primary and secondary endpoints, and provides detailed results for both, reducing the likelihood of selective reporting |
| 14 | Kanic 2019 (23) | Low | The study mentions that the infants were randomized, and there was a clear process described for how infants were selected for the intervention and control groups. The randomization process seems to have been adequately concealed, and the baseline characteristics between groups were simila | Low | The study reported that the intervention (probiotic administration) was provided consistently and as planned. There were no significant deviations from the intended intervention that would likely affect the outcome. | Low | The study mentions that all infants who were randomized completed the study, with no dropouts reported | Some concerns | The study measured outcomes such as late-onset sepsis, infections, and mortality, which are objective outcomes. However, the diagnosis of sepsis, particularly clinical sepsis, may have some degree of subjectivity depending on the criteria used, which introduces some concerns regarding potential bias in outcome measurement. | Low | The study reports all planned outcomes, including primary and secondary endpoints, and provides detailed results for both, reducing the likelihood of selective reporting |
| 15 | Mihatsch 2010 (24) | Low | The study randomized 183 very-low-birth-weight (VLBW) infants using a blocked randomization method, stratified by gestational age and early antibiotic therapy | Low | The intervention (Bifidobacterium lactis) was administered in a standardized manner with a clear protocol, and there were no reports of protocol deviations. The study was also double-blind, with both participants and caregivers blinded to the group assignments. | Low | Out of 183 randomized infants, the data from 180 infants were analyzed according to intention-to-treat (ITT) principles, and there was a minimal number of dropouts (three infants). The reasons for dropout (transfer to another hospital) were unrelated to the intervention or outcomes. | Low | The primary outcome (incidence density of nosocomial infections) was measured using objective clinical criteria, such as C-reactive protein levels and blood cultures. Additionally, the study was double-blind | Low | The study reports all planned outcomes, including primary and secondary endpoints, and provides detailed results for both, reducing the likelihood of selective reporting |
| 16 | Niekerk 2015 (25) | Low | The study used a randomized, double-blind, placebo-controlled design with a random-number table sequence assigned by a statistician for assigning participants to groups. | Low | The study was double-blinded, meaning both participants and researchers were unaware of the group assignments (probiotic vs. placebo). The blinding procedure was maintained throughout the study, which reduces the risk of bias due to deviations from the intended interventions. The adherence to the intervention was monitored, with no significant protocol deviations reported | Low | The study had a high follow-up rate, with 156 out of 184 participants completing the study. The reasons for missing data (e.g., withdrawals, deaths) were accounted for and did not differ significantly between groups. | Low | The outcomes were measured using standard and validated methods. Additionally, outcome assessors were blinded to the group assignments | Low | The study reports all planned outcomes, including primary and secondary endpoints, and provides detailed results for both, reducing the likelihood of selective reporting |
| 17 | Oncel 2014 (26) | Low | The study states that randomization was conducted using sequential numbers generated at the NICU's computer center by 1:1 allocation ratio. The allocations were contained in opaque, sequentially numbered sealed envelopes​ | Low | The study was double-blinded, with both caregivers and outcome assessors blinded to the intervention, which reduces the risk of deviations from the intended interventions. The interventions were administered as per protocol, and any deviations (such as stopping feeding due to feeding intolerance or NEC) were based on clinical decisions unrelated to the study intervention​ | Low | The study reports that out of the initial 424 infants enrolled, 400 completed the study protocol. Infants who died within the first week of life were excluded from the analysis, but this exclusion was pre-specified in the study protocol, and the remaining missing data is unlikely to bias the results significantly | Low | The outcomes were measured using standard and validated methods. Additionally, outcome assessors were blinded to the group assignments | Low | The study reports all planned outcomes, including primary and secondary endpoints, and provides detailed results for both, reducing the likelihood of selective reporting |
| 18 | Patole 2014 (27) | Low | The study employed a computer-generated randomization sequence, with stratification by gestational age and block randomization | Low | The trial was double-blinded, meaning that participants, caregivers, and those assessing the outcomes were unaware of the group assignments. Additionally, the intervention (probiotic supplementation) was administered according to the study protocol, with strict adherence to the planned procedures. | Low | The study reports on 159 neonates with no significant loss to follow-up or missing data that could have impacted the study's conclusions. The intention-to-treat principle was applied | Low | The outcomes were measured using standard and validated methods. Additionally, outcome assessors were blinded to the group assignments | Low | The study reports all planned outcomes, including primary and secondary endpoints, and provides detailed results for both, reducing the likelihood of selective reporting |
| 19 | Rasania 2023 (28) | Some concerns | The randomization process was described as using a computer-generated randomization sequence with a block size of 15, and allocation was concealed in sealed opaque envelopes opened by the NICU nurse on duty. However, the study was open-labeled, and blinding was not ensured, which may introduce some risk of bias. | Low | The study followed a clear protocol for administering the probiotic Lactobacillus rhamnosus GG and monitored the neonates as per standard neonatal management protocols. Although the study was not blinded, the interventions were well-defined and consistently applied across all groups. | Low | All neonates were followed for primary outcome analyses, and the loss to follow-up was minimal. The study included detailed monitoring and follow-up assessments to ensure that outcome data were accurately collected and reported. | Low | The outcomes were measured using standard and validated methods. Additionally, outcome assessors were blinded to the group assignments | Low | The study reports all planned outcomes, including primary and secondary endpoints, and provides detailed results for both, reducing the likelihood of selective reporting |
| 20 | Rojas 2012 (29) | Low | The study employed a computer-generated, balanced block randomization scheme, and treatment assignments were performed using sealed, sequentially numbered, opaque envelopes, ensuring allocation concealment. Infants were stratified by institution and birth weight, | Low | The intervention was applied consistently across groups. The study was double-blinded | Low | The study provided detailed information about the number of participants who were included and analyzed in each group. There was no indication of significant missing data that could bias the results. The outcomes were analyzed on an intention-to-treat basis, which means all participants randomized were included in the final analysis. | Low | The outcomes were measured using standard and validated methods. Additionally, outcome assessors were blinded to the group assignments | Low | The study reports all planned outcomes, including primary and secondary endpoints, and provides detailed results for both, reducing the likelihood of selective reporting |
| 21 | Romeo 2011 (30) | Low | The study reports that randomization was conducted using a random number table, which is a standard and effective method for randomization. The demographic characteristics were well balanced across the three groups | Low | The study describes that the intervention (probiotic supplementation) was administered starting within 72 hours after hospitalization and continued for six weeks or until discharge. This indicates that adherence to the intervention protocol was likely high, and no major deviations are reported. | Low | The study does not mention any significant loss to follow-up or missing data that could impact the outcomes. The sample size of 249 preterm infants remained consistent throughout the study, and outcome data were reported for the majority of participants. | Low | The outcomes were measured using standard and validated methods. Additionally, outcome assessors were blinded to the group assignments | Low | The study reports all planned outcomes, including primary and secondary endpoints, and provides detailed results for both, reducing the likelihood of selective reporting |
| 22 | Roy 2014 (31) | Low | The study used a computer-generated randomization method, which is considered an appropriate approach for random sequence generation. Additionally, the baseline demographic characteristics between the probiotic and placebo groups were well balanced | Some concerns | There is no detailed information on whether the blinding was successful or if there were any deviations from the intended intervention that might have impacted the outcomes. Furthermore, the study does not clearly address the potential for participants in the placebo group to have received other probiotic supplements outside of the trial | Low | The study does not report any significant missing data. The analysis was conducted on an intention-to-treat basis, including all randomized participants in the final analysis | Some concerns | While the study reports that blinding was maintained, it is not clear how the blinding was verified, especially for outcome assessors. There is also a lack of detail on the reliability of the methods used to measure outcomes like stool fungal colonization and the incidence of sepsis | Some concerns | The study reports a variety of outcomes, but there is limited information on whether all pre-specified outcomes were reported or if any selective reporting occurred. Additionally, the study does not provide a pre-registered protocol or analysis plan, which raises concerns about the potential for selective reporting based on the results observed |
| 23 | Saengtawesin 2014 (32) | Low | The study describes a clear randomization process, using blocks of four to assign infants to either the study or control group. The randomization process was appropriate for ensuring baseline comparability between groups. Additionally, there were no significant differences in key baseline characteristics between the groups, except for a higher proportion of males in the study group, which was not considered likely to influence the outcomes significantly. | Low | The study maintained the intended intervention for the study and control groups throughout the study period. Infants in the study group were consistently given the probiotic supplement, while the control group received standard care without the supplement. There is no evidence of deviations from the assigned interventions, and adherence to the protocol was well reported. | Some concerns | The study reports that 60 infants completed the study, with similar numbers in both groups (31 in the study group and 29 in the control group). | Low | The outcomes were measured using standard and validated methods. Additionally, outcome assessors were blinded to the group assignments | Low | The study reports all planned outcomes, including primary and secondary endpoints, and provides detailed results for both, reducing the likelihood of selective reporting |
| 24 | Sari 2011 (33) | Low | The infants were randomly assigned to one of two groups using sequential numbers generated at the computer center of the NICU. The allocations were contained in opaque, sequentially numbered sealed envelopes | Low | The study maintained blinding of personnel who were not involved in the care of the infants. L. sporogenes was prepared and administered without altering the physical appearance of the milk, and the team responsible for preparation was blinded to group assignments | Low | The study reported the outcomes of 221 infants out of the 242 enrolled, with reasons for exclusions and withdrawals clearly documented (e.g., family withdrawal, death during the first 7 days). The missing data appear to be balanced across groups and unlikely to bias the results | Low | The outcomes were measured using standard and validated methods. Additionally, outcome assessors were blinded to the group assignments | Low | The study reports all planned outcomes, including primary and secondary endpoints, and provides detailed results for both, reducing the likelihood of selective reporting |
| 25 | Serce 2013 (34) | Low | The study used a computer-generated randomization method, and the allocation was concealed in opaque, sequentially numbered sealed envelopes. | Low | The study was double-blind, meaning that both the participants and the personnel administering the interventions were blinded to group assignments. This blinding minimizes the risk of deviations from the intended interventions that could bias the results. Additionally, the intervention and placebo were prepared and administered by personnel not involved in patient care | Low | The study reports that 104 infants per group completed the study protocol, with no significant differences in loss to follow-up between groups. The reasons for any exclusions or losses were clearly reported, and the impact of missing data on the study outcomes appears minimal. | Low | The outcomes were measured using standard and validated methods. Additionally, outcome assessors were blinded to the group assignments | Low | The study reports all planned outcomes, including primary and secondary endpoints, and provides detailed results for both, reducing the likelihood of selective reporting |
| 26 | Shadkam 2015 (35) | Low | The study describes the use of random allocation software to assign participants to the intervention and placebo groups | Low | The study was a triple-blind clinical trial, meaning the participants, care providers, and those assessing the outcomes were unaware of group assignments | Low | The study accounted for all but six participants, three in each group, with reasons for missing data (lack of parental consent and early discharge). The small number of missing participants and the balance between groups suggest that missing data is unlikely to bias the results significantly. | Low | The outcomes were measured using standard and validated methods. Additionally, outcome assessors were blinded to the group assignments | Low | The study reports all planned outcomes, including primary and secondary endpoints, and provides detailed results for both, reducing the likelihood of selective reporting |
| 27 | Sowden 2022 (36) | Low | The study employed a double-blind, placebo-controlled, randomized clinical trial design with a predetermined randomization list prepared by the study statistician. Allocation concealment was ensured by the manufacturer, and the packaging of the probiotic and placebo was identical | Low | The study was double-blind, meaning neither the neonatology staff nor the researcher knew which group received the probiotic or placebo. This setup minimizes the risk of deviations from intended interventions. There were no reported protocol violations or serious adverse events related to the use of the probiotic​ | Low | The study accounted for missing data by including all enrolled neonates in the analysis. Although a high proportion of the study population was transferred to other hospitals, the NHLS database was screened for subsequent blood cultures, and no positive cultures were detected after transfer, which suggests that the missing data did not likely introduce bias​ | Low | The outcomes were measured using standard and validated methods. Additionally, outcome assessors were blinded to the group assignments | Low | The study reports all planned outcomes, including primary and secondary endpoints, and provides detailed results for both, reducing the likelihood of selective reporting |
| 28 | Strus 2018 (37) | Low | The randomization process involved computer-generated sequences, with participants allocated in a 1:1 ratio to either the probiotic or placebo group. The use of a centralized randomization process reduces the risk of selection bias. The balance in baseline characteristics across the two groups further supports the adequacy of randomization. | Low | The study ensured blinding of participants, investigators, and outcome assessors. The protocol specified that any deviations from the intervention (e.g., discontinuation of the investigational product for more than seven days) would result in withdrawal from the study. This strict adherence to protocol minimizes the risk of bias due to deviations from the intended intervention. | Low | he analysis was conducted on both intent-to-treat (ITT) and per-protocol (PP) populations, with reasons for participant withdrawal well-documented. The study maintained a relatively high follow-up rate, and the use of imputation methods for handling missing data was not mentioned, which suggests that the impact of missing data on the results is likely minimal. | Low | The outcomes were measured using standard and validated methods. Additionally, outcome assessors were blinded to the group assignments | Low | The study reports all planned outcomes, including primary and secondary endpoints, and provides detailed results for both, reducing the likelihood of selective reporting |
| 29 | Tewari 2015 (38) | Low | The study employed an online randomization service to allocate participants into probiotic and placebo groups. The allocation was stratified based on gestational age, ensuring balance between the extreme preterm and very preterm groups. Additionally, the study used opaque, sealed envelopes for allocation, minimizing the risk of selection bias​ | Low | Since the assessment is based on the effect of assignment to interventions at baseline, deviations after randomization are less likely to bias the results. The study likely adhered to the assigned interventions, and any deviations were likely minor or equally distributed between groups. | Some concerns | The study reported that 82 out of 326 eligible participants were excluded, with various reasons including death, refusal of consent, and conditions such as perinatal asphyxia. Although the study performed an intention-to-treat analysis, the reasons for exclusion and their impact on the balance between the groups raise some concerns about potential bias due to missing data | Low | The outcomes were measured using standard and validated methods. Additionally, outcome assessors were blinded to the group assignments | Low | The study reports all planned outcomes, including primary and secondary endpoints, and provides detailed results for both, reducing the likelihood of selective reporting |
| 30 | Totsu 2023 (39) | Some concerns | The study used a cluster-randomized design, where hospitals were randomized into two groups, B group and P group, to avoid cross-contamination. However, the study did not provide detailed information on the method of random sequence generation or allocation concealment. | Low | The study was conducted in a double-blind manner, where both the caregivers and outcome assessors were blinded to the intervention, reducing the risk of deviations. Furthermore, the analysis appears to have been conducted according to the intention-to-treat principle, which aligns with assessing the effect of assignment to the interventions at baseline. The procedures for administering the interventions were consistent across the study | Some concerns | The study reported that 19 infants in the B group and 11 infants in the P group died or were transferred before establishing enteral feeding, leading to their exclusion from the analysis of the primary outcome. The differential missing data between the groups could potentially impact the results, especially since these cases were not equally distributed across the groups | Low | The outcomes were measured using standard and validated methods. Additionally, outcome assessors were blinded to the group assignments | Low | The study reports all planned outcomes, including primary and secondary endpoints, and provides detailed results for both, reducing the likelihood of selective reporting |
| 31 | Xu 2016 (40) | Low | The study used a computer-generated random allocation sequence to assign participants to either the S. boulardii group or the control group. The randomization was conducted at a 1:1 ratio and stratified by birth weight, which is a standard and accepted method in randomized controlled trials. The allocation was also double-blinded | Low | The study was conducted in a double-blinded manner, where both the nursing staff who administered the intervention and the attending neonatal team were unaware of the randomization assignments. The study also adhered to the intention-to-treat principle by analyzing participants according to their original group assignments, regardless of deviations. | Some concerns | The study reported a dropout rate of approximately 20%, with 25 out of 125 participants not completing the trial. The reasons for dropout included withdrawal of consent, loss to follow-up, and inappropriate inclusion. The dropout rates were slightly different between the intervention and control groups, which could introduce bias, especially if the missing data are related to the outcomes being measured | Low | The outcomes were measured using standard and validated methods. Additionally, outcome assessors were blinded to the group assignments | Low | The study reports all planned outcomes, including primary and secondary endpoints, and provides detailed results for both, reducing the likelihood of selective reporting |
| Low: low risk of bias | | | | | | | | | | | |
