# Supplementary material for: Probiotics for preventing neonatal sepsis in preterm neonates: a systematic review and meta-analysis for clinical practice
Source: Epidemiol Health. 2025 Sep 3;47:e2025051. doi: 10.4178/epih.e2025051 (PMC12869123; doi:10.4178/epih.e2025051)
Supplement: Supplementary Material 4. — Summary of findings and certainty assessment (GRADE) [file epih-47-e2025051-Supplementary-4.docx]

## Supplementary Material 4. Summary of findings and certainty assessment (GRADE)

| **Probiotics compared to control for prevention of neonatal sepsis** | | | | | | | | | | | |
| --- | --- | --- | --- | --- | --- | --- | --- | --- | --- | --- | --- |
| **Certainty assessment** | | | | | | | **Summary of findings** | | | | |
| **Participants (studies) Follow-up** | **Risk of bias** | **Inconsistency** | **Indirectness** | **Imprecision** | **Publication bias** | **Overall certainty of evidence** | **Study event rates (%)** | | **Relative effect (95% CI)** | **Anticipated absolute effects** | |
|  |  |  |  |  |  |  | **With control** | **With probiotics** |  | **Risk with control** | **Risk difference with probiotics** |
| **Late-onset sepsis** | | | | | | | | | | | |
| 8040 (30 RCTs) | not serious | serious^a^ | not serious | not serious | none | ⨁⨁⨁◯ Moderate^a^ | 653/3959 (16.5%) | 570/4081 (14.0%) | **RR 0.83** (0.72 to 0.95) | **High** | |
|  |  |  |  |  |  |  |  |  |  | 830 per 1,000 | **141 fewer per 1,000** (from 232 fewer to 42 fewer) |
| **Late-onset sepsis; using Lactobacillus sp.** | | | | | | | | | | | |
| 2113 (8 RCTs) | not serious | not serious | not serious | serious^b^ | none | ⨁⨁⨁◯ Moderate^b^ | 124/1060 (11.7%) | 92/1053 (8.7%) | **RR 0.74** (0.53 to 1.04) | 124/1060 (11.7%) | **30 fewer per 1,000** (from 55 fewer to 5 more) |
| **Late-onset sepsis; using Bifidobacterium sp.** | | | | | | | | | | | |
| 2288 (6 RCTs) | not serious | not serious | not serious | serious^b^ | none | ⨁⨁⨁◯ Moderate^b^ | 147/1136 (12.9%) | 156/1152 (13.5%) | **RR 1.06** (0.83 to 1.36) | 147/1136 (12.9%) | **8 more per 1,000** (from 22 fewer to 47 more) |
| **Late-onset sepsis; using Saccharomyces sp.** | | | | | | | | | | | |
| 579 (3 RCTs) | not serious | not serious | not serious | serious^b^ | none | ⨁⨁⨁◯ Moderate^b^ | 52/289 (18.0%) | 43/290 (14.8%) | **RR 0.83** (0.57 to 1.19) | 52/289 (18.0%) | **31 fewer per 1,000** (from 77 fewer to 34 more) |
| **Late-onset sepsis; using multistrain** | | | | | | | | | | | |
| 2816 (12 RCTs) | not serious | serious^c^ | not serious | not serious | none | ⨁⨁⨁◯ Moderate^c^ | 305/1353 (22.5%) | 259/1463 (17.7%) | **RR 0.76** (0.61 to 0.95) | 305/1353 (22.5%) | **54 fewer per 1,000** (from 88 fewer to 11 fewer) |
| **Late-onset sepsis; using high dosage (>10^9 CFU/day)** | | | | | | | | | | | |
| 2590 (15 RCTs) | not serious | not serious | not serious | serious^b^ | none | ⨁⨁⨁◯ Moderate^b^ | 241/1275 (18.9%) | 215/1315 (16.3%) | **RR 0.89** (0.74 to 1.08) | 241/1275 (18.9%) | **21 fewer per 1,000** (from 49 fewer to 15 more) |
| **Late-onset sepsis; using low dosage (≤10^9 CFU/day)** | | | | | | | | | | | |
| 3668 (13 RCTs) | not serious | serious^d^ | not serious | not serious | none | ⨁⨁⨁◯ Moderate^d^ | 280/1791 (15.6%) | 227/1877 (12.1%) | **RR 0.72** (0.56 to 0.91) | 280/1791 (15.6%) | **44 fewer per 1,000** (from 69 fewer to 14 fewer) |
| **Mortality** | | | | | | | | | | | |
| 6815 (24 RCTs) | not serious | not serious | not serious | serious^b^ | none | ⨁⨁⨁◯ Moderate^b^ | 242/3346 (7.2%) | 206/3469 (5.9%) | **RR 0.84** (0.70 to 1.00) | 242/3346 (7.2%) | **12 fewer per 1,000** (from 22 fewer to 0 fewer) |
| **Mortality; using Lactobacillus sp.** | | | | | | | | | | | |
| 1139 (5 RCTs) | not serious | not serious | not serious | serious^b^ | none | ⨁⨁⨁◯ Moderate^b^ | 39/570 (6.8%) | 28/569 (4.9%) | **RR 0.72** (0.45 to 1.16) | 39/570 (6.8%) | **19 fewer per 1,000** (from 38 fewer to 11 more) |
| **Mortality; using Bifidobacterium sp.** | | | | | | | | | | | |
| 1693 (3 RCTs) | not serious | serious^e^ | not serious | serious^b^ | none | ⨁⨁◯◯ Low^b,e^ | 69/850 (8.1%) | 59/843 (7.0%) | **RR 0.87** (0.62 to 1.21) | 69/850 (8.1%) | **11 fewer per 1,000** (from 31 fewer to 17 more) |
| **Mortality; using Saccharomyces sp.** | | | | | | | | | | | |
| 479 (2 RCTs) | not serious | not serious | not serious | serious^f^ | none | ⨁⨁⨁◯ Moderate^f^ | 9/240 (3.8%) | 10/239 (4.2%) | **RR 1.12** (0.46 to 2.70) | 9/240 (3.8%) | **5 more per 1,000** (from 20 fewer to 64 more) |
| **Mortality; using multistrain** | | | | | | | | | | | |
| 2756 (11 RCTs) | not serious | not serious | not serious | serious^b^ | none | ⨁⨁⨁◯ Moderate^b^ | 98/1324 (7.4%) | 86/1432 (6.0%) | **RR 0.83** (0.63 to 1.10) | 98/1324 (7.4%) | **13 fewer per 1,000** (from 27 fewer to 7 more) |
| **Mortality; using high dosage (>10^9 CFU/day)** | | | | | | | | | | | |
| 1952 (10 RCTs) | not serious | not serious | not serious | serious^b^ | none | ⨁⨁⨁◯ Moderate^b^ | 56/958 (5.8%) | 41/994 (4.1%) | **RR 0.72** (0.49 to 1.06) | 56/958 (5.8%) | **16 fewer per 1,000** (from 30 fewer to 4 more) |
| **Mortality; using low dosage (≤10^9 CFU/day)** | | | | | | | | | | | |
| 3009 (10 RCTs) | not serious | not serious | not serious | serious^b^ | none | ⨁⨁⨁◯ Moderate^b^ | 94/1460 (6.4%) | 79/1549 (5.1%) | **RR 0.82** (0.61 to 1.10) | 94/1460 (6.4%) | **12 fewer per 1,000** (from 25 fewer to 6 more) |
| **Length-of-stay** | | | | | | | | | | | |
| 5952 (17 RCTs) | not serious | very serious^g^ | not serious | not serious | none | ⨁⨁◯◯ Low^g^ | 2974 | 2978 | - | 2974 | MD **3.72 days fewer** (5.41 fewer to 2.03 fewer) |
| **Length-of-stay; using Lactobacillus sp.** | | | | | | | | | | | |
| 1832 (6 RCTs) | not serious | very serious^h^ | not serious | not serious | none | ⨁⨁◯◯ Low^h^ | 919 | 913 | - | 919 | MD **5.34 days fewer** (8.47 fewer to 2.2 fewer) |
| **Length-of-stay; using Bifidobacterium sp.** | | | | | | | | | | | |
| 1822 (4 RCTs) | not serious | very serious^i^ | not serious | serious^j^ | none | ⨁◯◯◯ Very low^i,j^ | 916 | 906 | - | 916 | MD **3.71 days fewer** (12.14 fewer to 4.72 more) |
| **Length-of-stay; using Saccharomyces sp.** | | | | | | | | | | | |
| 604 (3 RCTs) | not serious | very serious^k^ | not serious | serious^j^ | none | ⨁◯◯◯ Very low^j,k^ | 302 | 302 | - | 302 | MD **1.65 days fewer** (6.65 fewer to 3.34 more) |
| **Length-of-stay; using multistrain** | | | | | | | | | | | |
| 1411 (3 RCTs) | not serious | very serious^l^ | not serious | serious^j^ | none | ⨁◯◯◯ Very low^j,l^ | 707 | 704 | - | 707 | MD **2.39 days fewer** (5.22 fewer to 0.43 more) |
| **Length-of-stay; using high dosage (>10^9 CFU/day)** | | | | | | | | | | | |
| 1832 (10 RCTs) | not serious | very serious^m^ | not serious | not serious | none | ⨁⨁◯◯ Low^m^ | 903 | 929 | - | 903 | MD **3.7 days fewer** (5.82 fewer to 1.58 fewer) |
| **Length-of-stay; using low dosage (≤10^9 CFU/day)** | | | | | | | | | | | |
| 2810 (7 RCTs) | not serious | very serious^n^ | not serious | not serious | none | ⨁⨁◯◯ Low^n^ | 1411 | 1399 | - | 1411 | MD **4.63 days fewer** (8.05 fewer to 1.22 fewer) |

**CI:** confidence interval; **MD:** mean difference; **RR:** risk ratio

#### Explanations

a. Serious unexplained inconsistency (moderate heterogeneity I2 = 34%, P value [P = 0.006], point estimates and confidence intervals vary considerably)

b. Serious imprecision: Wide confidence interval includes both clinically important thresholds (RR < 0.75 or RR > 1.25) and no effect (RR = 1.0).

c. Serious unexplained inconsistency (moderate heterogeneity I2 = 50%, P value [P = 0.02], point estimates and confidence interval vary considerably)

d. Serious unexplained inconsistency (moderate heterogeneity I2 = 44%, P value [P = 0.007], point estimates and confidence interval vary inconsiderably)

e. Serious unexplained inconsistency (moderate heterogeneity I2 = 58%, P value [P = 0.40], point estimates and confidence interval vary inconsiderably)

f. Very serious imprecision: Very wide confidence interval spans substantial benefit (RR < 0.75), substantial harm (RR > 1.25), and no effect (RR = 1.0).

g. Very serious unexplained inconsistency (large heterogeneity I2 = 75%, P value [P <0.001], point estimates and confidence interval vary considerably)

h. Very serious unexplained inconsistency (large heterogeneity I2 = 77%, P value [P < 0.001], point estimates and confidence interval vary considerably)

i. Very serious unexplained inconsistency (large heterogeneity I2 = 78%, P value [P = 0.39], point estimates and confidence interval vary considerably)

j. Serious imprecision for continuous outcome: P value >0.05

k. Very serious unexplained inconsistency (large heterogeneity I2 = 75%, P value [P = 0.52], point estimates and confidence interval vary considerably)

l. Very serious unexplained inconsistency (large heterogeneity I2 = 61%, P value [P = 0.10], point estimates and confidence interval vary considerably)

m. Very serious unexplained inconsistency (large heterogeneity I2 = 65%, P value [P < 0.001], point estimates and confidence interval vary considerably)

n. Very serious unexplained inconsistency (large heterogeneity I2 = 81%, P value [P = 0.008], point estimates and confidence interval vary considerably)
